# Thiol–Yne Photocurable Isosorbide-Derived Networks: Formulation and 3D Printing

**DOI:** 10.1021/acssuschemeng.5c13600

**Published:** 2026-02-04

**Authors:** Dumitru Moraru, Giacomo Trapasso, Davide Dalla Torre, Thomas Griesser, Fabio Aricò, Marco Sangermano

**Affiliations:** † Dipartimento di Scienza Applicata e Tecnologia, Politecnico di Torino, 10129 Torino, Italy; ‡ Department of Environmental Sciences, Informatics and Statistics, 19032 Ca’ Foscari University of Venice, Via Torino 155, 30172 Venezia Mestre, Italy; § Institute of Chemistry of Polymeric Materials, Technical University of Leoben, Otto Glöckelstrasse 2, 8700 Leoben, Austria

**Keywords:** isosorbide, biobased alkyne monomers, thiol−yne photopolymerization, UV-curing, 3D printing

## Abstract

The present work reports for the first time thiol–yne photoresins prepared from novel alkyne derivatives of isosorbide and its epimers, isomannide and isoidide. Isosorbide was selected as a key biobased monomer for this study in consideration of its unique rigid V-shaped structure and peculiar reactivity, as well as for the growing interest in this cyclic sugar due to its numerous industrial applications in polymer science. Dialkyl carbonate chemistry was used for the preparation of dipropargyl derivatives of isosorbide and its epimers via alkoxycarbonylation reaction conducted under mild conditions using catalytic amounts of 1,5,7-triazabicyclo[4.4.0]­dec-5-ene (TBD). Dialkyne monomers were then employed to produce biobased thiol–yne photoresins, formulated using trimethylolpropane tris­(3-mercaptopropionate) as a trifunctional thiol. The photopolymerization behavior was investigated by real-time Fourier-transform infrared spectroscopy and differential scanning calorimetry (DSC) to assess conversion efficiency and reaction kinetics. The resulting networks were characterized by DSC and dynamic mechanical thermal analysis. Furthermore, residual thiol groups enabled surface modification with poly­(ethylene glycol) methacrylate (PEGMA) to enhance hydrophilicity, as confirmed by contact angle measurements. Finally, the optimized isosorbide-based network formulation was successfully printed by digital light processing, achieving accurate 3D-printed structures.

## Graphical abstract

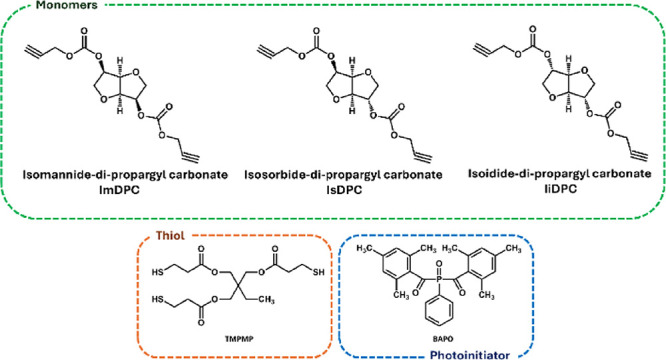

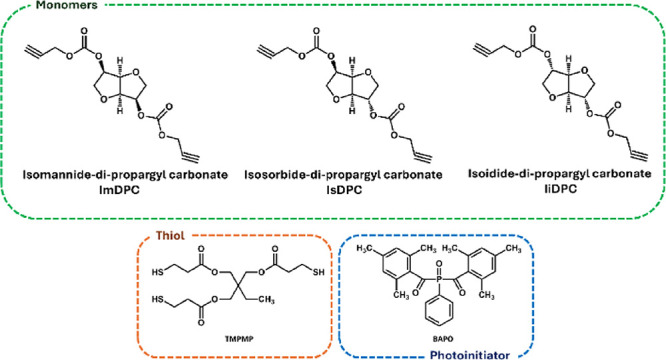

## 1 Introduction

Among the various techniques available to produce biobased polymers, thiol–ene and thiol–yne (TY) click chemistry have been increasingly explored due to their high versatility. This methodology holds significant promise for material synthesis and modification, offering distinct advantages in macromolecular engineering, polymer science, and advanced applications. The TY process typically proceeds via a two-step addition reaction, where a thiol first adds to an alkyne to form a vinyl sulfide intermediate, followed by the addition of a second thiol molecule to the so-formed vinyl sulfide.^1^ The alkyne functional group can sequentially react with two thiol groups, which inherently enables higher cross-link densities than those achievable through thiol–ene reactions (Scheme 1).^2,3^

**Scheme 1:**
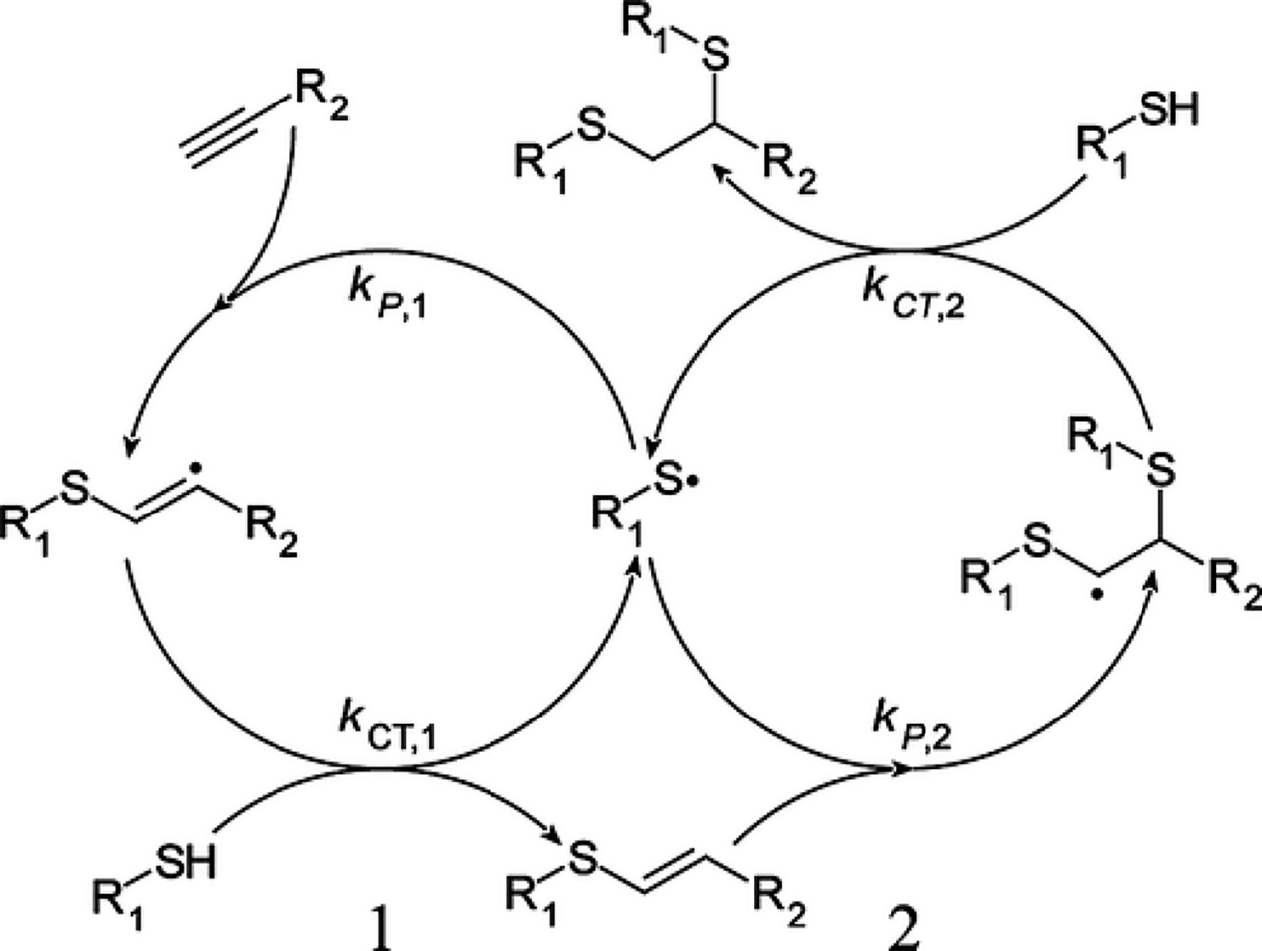
Simplified Mechanism of Radical TY Photopolymerization

TY photopolymerization, due to its step-growth mechanism, exhibits a delayed gel-point conversion that leads to lower shrinkage stress and the formation of more homogeneous networks with fewer unreacted compounds, contrasting sharply with chain-growth polymerizations.^4^ The high conversion achieved at the gel point, compared to acrylates, significantly reduces the shrinkage stress in the final material.^5–7^

Thiol–ene and TY click chemistry have emerged as powerful strategies in synthetic chemistry, offering several key benefits including mild reaction conditions, rapid kinetics, broad applicability, and high conversion efficiencies and yields.^8–12^ Moreover, TY systems offer notable advantages because their surface properties can be tailored either through simple postsynthetic modifications or by using off-stoichiometric formulations that generate an excess of thiol or alkyne functional groups. This tunability renders them highly attractive for a wide range of applications.

Thiol–ene-based resins are well recognized for their minimal warping and consistent curing behavior. Similarly, TY systems retain these advantages while offering the possibility of higher cross-link density when needed. This constitutes an important feature for additive manufacturing (AM) methods such as vat photopolymerization, including stereolithography (SLA) and digital light processing (DLP). Recently, a review reported the exploitation of thiol–ene and TY reactions in visible-light activation, which is important for designing DLP/SLA-TY resins that cure under visible light.^13^ Similarly, another review focused on thiol–ene and TY for AM, covering kinetics, oxygen tolerance, step-growth benefits, and examples of printable formulations.^14^

Furthermore, Trujillo-Lemon et al. demonstrated that TY formulations can be designed to polymerize under visible light, enabling the DLP printing of intricate, high-resolution structures. Because curing follows a step-growth mechanism, the process proceeds uniformly throughout the material, preventing issues like oxygen inhibition or vitrification and ensuring accurate dimensional fidelity in 3D-printed components.^2,15,16^

Moreover, TY photopolymer systems represent promising candidates for the fabrication of medical materials by using UV-based 3D-printing technologies. In particular, alkyne ethers^17^ and alkyne carbonates^18–20^ exhibit low monomer cytotoxicity, suitable photoreactivity, high monomer conversions, and excellent mechanical performance in terms of toughness in the cured state.

Furthermore, degradable polymer networks can be generated either by exploiting the ester moieties present in thiol monomers^19^ or by employing degradable, alkyne-functionalized amino acid phosphorodiamidates^21,22^ and carbonate-based alkyne monomers,^23^ making these systems particularly attractive for tissue-engineering applications. Notably, the degradation rate of such materials can be tuned by selection of the monomers and their relative ratios.

In line with sustainability trends, researchers have explored TY monomers derived from natural products and recycled materials. Many traditional thiol monomers, e.g., pentaerythritol tetrakis­(3-mercaptopropionate)—PETMP and trimethylolpropane tris­(3-mercaptopropionate)—TMPMP are petroleum-based, but recent works have shown renewable alternatives. One example is the exploitation of free thiol generation under UV of lipoic acid methyl ester (a naturally occurring cyclic disulfide) in combination with various alkynes to create high-sulfur networks for optical applications. This bioderived dithiolane provided an in situ source of thiols upon ring opening, enabling a solvent-free TY photopolymerization.^24^ Similarly, different biobased alkyne monomers have been synthesized to be exploited in the UV-curing process. Some examples in the literature focused on the production of different biobased photocurable hydrogels employing alkyne-modified alginate,^25^ and thermosets made by alkyne-functionalized vegetable-oil derivatives.^26^ Moreover, in the field of biofabrication and tissue engineering, researchers have developed tyrosine-based photoresins for 3D printing of scaffolds that are biocompatible and even biodegradable, enabling their use in in vitro ovarian follicle culture.^27^

Despite these examples, the synthesis and investigation of biobased monomers suitable for TY polymerization remain scarce. In this view, we decided to explore for the first time TY photoresins prepared from novel alkyne derivatives of isosorbide and its epimers, isomannide and isoidide.

Isosorbide, derived from nonedible cellulosic biomass, was selected for this study because—in line with the United Nations’ recent sustainability goals—it is anticipated to become a key biobased platform molecule for the production of fine chemicals and biodegradable polymers.^28–32^ Isosorbide, readily obtained by the double dehydration of D-sorbitol,^33–35^ has attracted significant scientific interest due to its rigid V-shaped structure coupled with a distinctive chemical reactivity. This cyclic sugar is characterized by the peculiar behavior of its two hydroxyl moieties, whose reactivity is enhanced compared to other secondary alcohols.^36,37^ The different spatial orientations of the two hydroxyl groups render the *endo* OH, pointing inside the bicyclic cavity, less reactive compared to the *exo* one, directed outside the sugar cavity. This behavior has been proven by exploiting the reactivity of the two isosorbide epimers, isomannide and isoidide, whose OH groups are both *endo* and *exo*, respectively.^38^

The relevance of isosorbide as biobased platform chemical is underscored by its use as a substrate for the production of pharmaceuticals,^38,39^ solvents,^40–42^ additives, plasticizers,^43,44^ surfactants,^45^ and polymers (Figure 1).^30,32,46,47^ Interestingly, over the years, it has been demonstrated that dialkyl carbonates (DACs)^36^ can serve as green media and/or reagents for the chlorine-free derivatization of isosorbide, affording both biobased solvents, i.e., dimethyl isosorbide (DMI) and monomers, including alkoxycarbonate derivatives, for polymer synthesis.^40–42^

**Figure 1:**
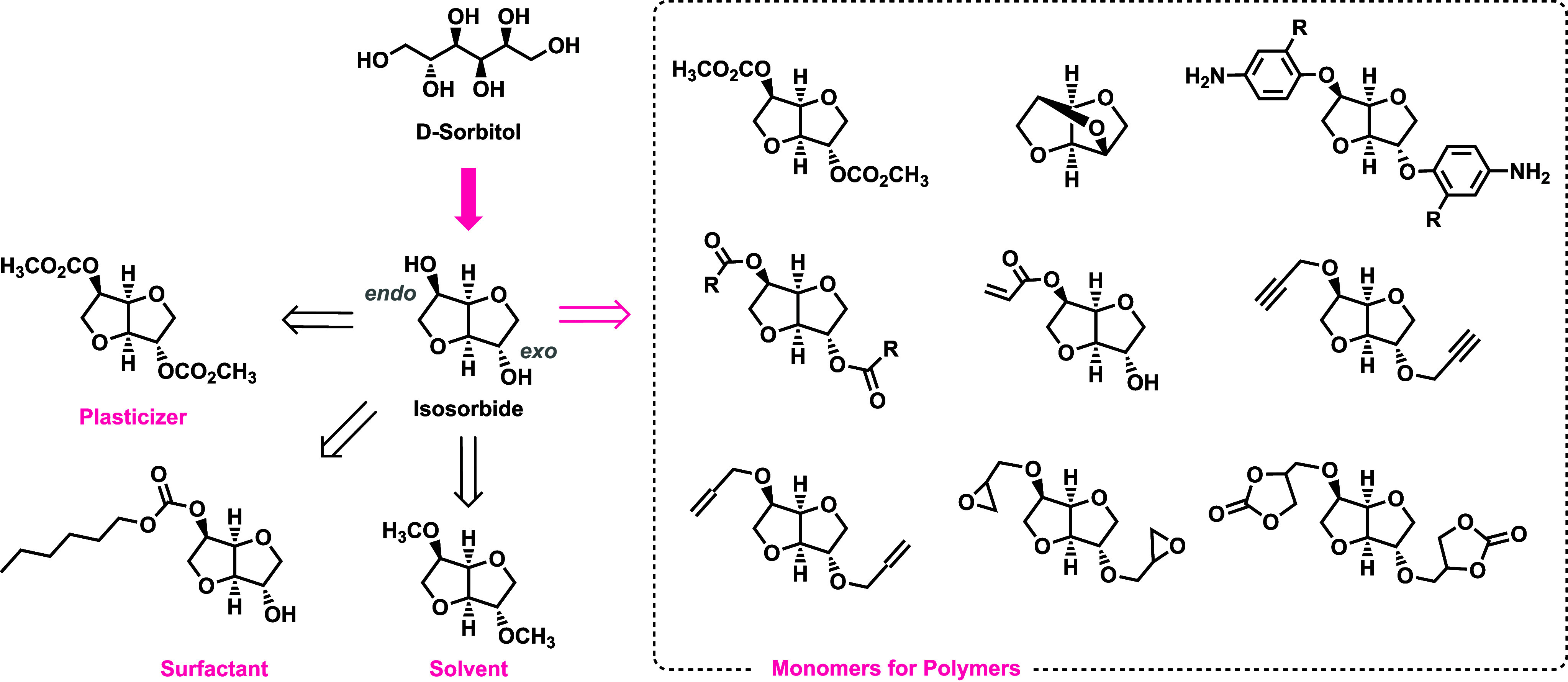
Isosorbide as biobased platform chemical.

As stated above, this research focuses on the preparation of novel alkyne derivatives of isosorbide and its epimers via DAC chemistry, namely, isosorbide dipropargyl carbonate (IsDPC), isomannide dipropargyl carbonate (ImDPC), and isoidide dipropargyl carbonate (IiDPC). The new alkyne monomers were subsequently employed in TY UV-curable formulations using TMPMP as the thiol component. Although this thiol is currently of fossil origin, it could, in principle, be obtained from renewable sources through the appropriate thiol functionalization.

The photopolymerization process of the alkyne derivatives of the three cyclic sugars was investigated by Fourier-transform infrared spectroscopy (FT-IR) and photodifferential scanning calorimetry (photo-DSC). The resulting cross-linked networks were characterized by differential scanning calorimetry (DSC) and dynamic mechanical thermal analysis (DMTA) to assess their thermal and thermo-mechanical properties. Furthermore, a UV-induced postgrafting process was demonstrated by exploiting residual unreacted thiol groups to tailor the surface characteristics of the cross-linked materials.

Optimized printing parameters were established, enabling the successful 3D printing of complex geometries with a high resolution. These results demonstrate the feasibility of utilizing TY biobased formulations for advanced 3D printing applications by combining renewable monomer sources with efficient photopolymerization chemistry.

## 2 Experimental Section

### 2.1 Materials

The following reagents were purchased from Sigma-Merck and employed without any further purification: isosorbide, isomannide, isoidide, trimethylolpropane tris­(3-mercaptopropionate)—TMPMP, phenylbis­(2,4,6-trimethylbenzoyl)­phosphine oxide—BAPO, poly­(ethylene glycol) methacrylate—PEGMA—average Mn 360, methacrylate. Monomer syntheses have been conducted in a silicon oil bath or in a Drysyn instrument at the required temperature.

### 2.2 Synthesis of Isosorbide, Isomannide, and Isoidide Monomers

The synthesis of isosorbide dipropargyl carbonate (IsDPC), isomannide dipropargyl carbonate (ImDPC), and isoidide dipropargyl carbonate (IiDPC) was performed by reacting the corresponding sugar with dipropargyl carbonate. The structures of these monomers are depicted in Scheme 2. Based on the NMR limit of detection, the purity of the isolated monomers can be estimated to be between 95 and 98%.

**Scheme 2:**
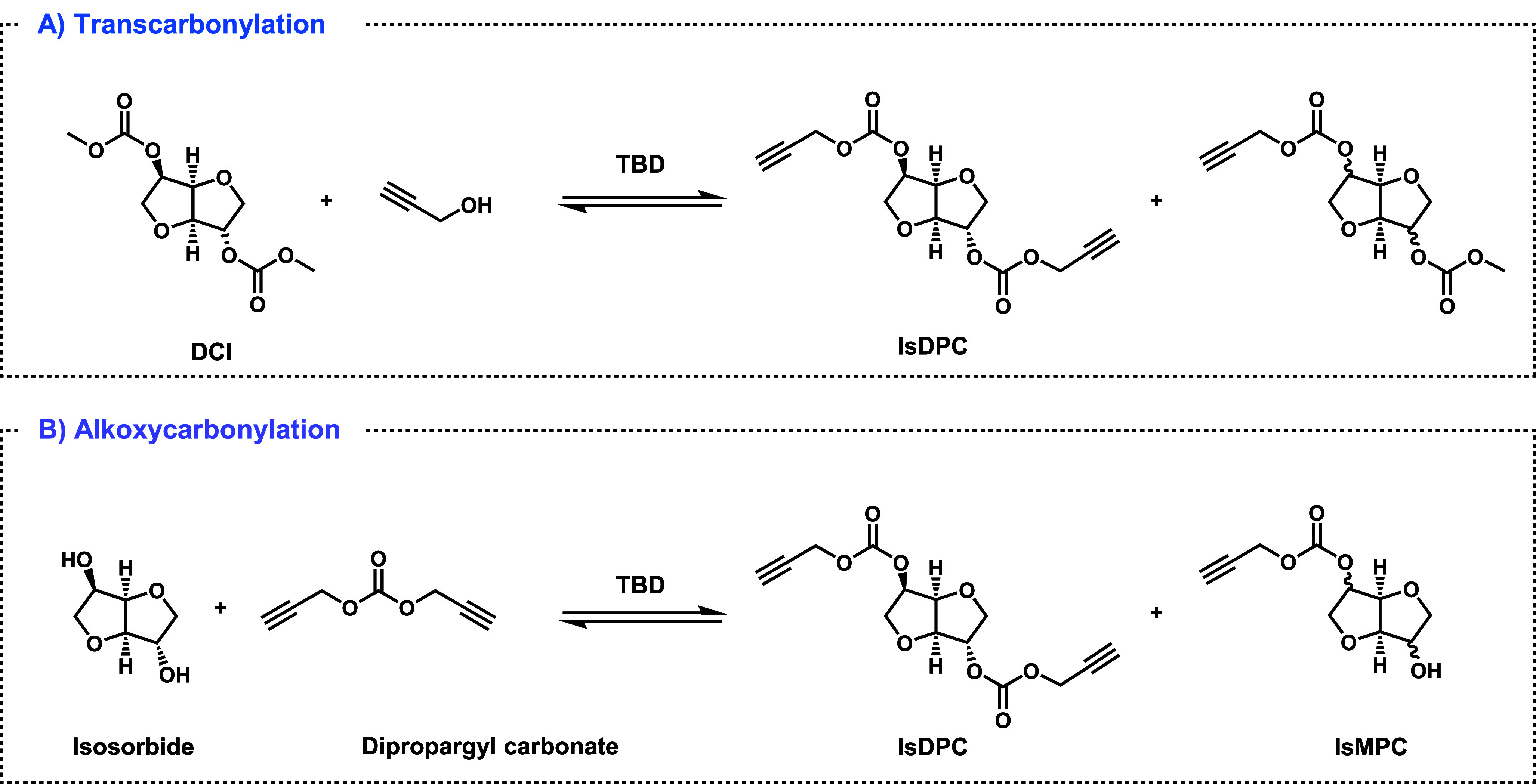
Synthetic Pathways Explored for IsDPC

#### 2.2.1 Synthesis of Dipropargyl Carbonate

Following an adapted literature procedure for DAC synthesis,^42^ DMC (10.0 mL, 0.12 mol, 1.0 mol eq.), propargyl alcohol (35.0 mL, 0.61 mol, 5.0 mol eq.), and TBD (0.33 g, 2.40 mmol, 0.02 mol eq.) were added in a 100 mL two-neck round-bottom flask equipped with a water condenser and a magnetic stirrer. The temperature was kept at DMC reflux conditions (95–100 °C) for 4 h. Afterward, the condenser was swapped with a Dean–Stark apparatus, and a nitrogen flow was started to remove methanol formed as a side product. The reaction was monitored via silica TLC using CH_2_Cl_2_ as an eluent phase. At the end of the reaction time, the reaction was cooled down, propargyl alcohol was distilled at 50 °C under vacuum (*p* = 30 mbar; 10–15 mL recovered as a transparent liquid), and the residue was filtered on a silica pad and washed with EtOAc (20.0 mL × 3). Dipropargyl carbonate was obtained as an orange liquid in 41% yield (6.78 g).

^1^H NMR (400 MHz, CDCl_3_) δ (ppm) = 4.78–4.79 (d, 4H), 2.56–2.57 (t, 2H).

^13^C NMR (100 MHz, CDCl_3_) δ (ppm) = 154.0, 76.6, 76.0, 55.7.

HR-MS: *m*/*z* [M + Na]^+^ calc. for [C_7_H_6_O_3_+Na]^+^: 161.0226; found: 161.0209.

#### 2.2.2 Synthesis of Isosorbide Dipropargyl Carbonate—IsDPC

In a 100 mL two-neck round-bottom flask equipped with a Dean–Stark apparatus, a water condenser, and a magnetic stir bar, isosorbide (4.00 g, 27.30 mmol, 1.0 mol equiv) was dissolved in dipropargyl carbonate (38.30 g, 0.27 mol, 10.0 mol equiv) in the presence of TBD (0.036 g, 0.27 mmol, 0.01 mol eq.). The reaction mixture was heated at 100 °C for 2 h under magnetic stirring and a nitrogen flow. Afterward, the hot reaction mixture was filtered through a silica pad on a Gooch filter under vacuum, and the silica pad was washed with EtOAc (20.0 mL × 3) to maximize product recovery. All the liquid was collected in a round-bottom flask and cooled to room temperature. Ethyl acetate was removed by rotary evaporation under vacuum, and the dipropargyl carbonate excess was removed from the reaction mixture through a fractional distillation procedure under vacuum (*T*_(ext)_ = 70–75 °C, *p* = 30 mbar) as a transparent liquid and reused in subsequent trials. The product was obtained as pure via silica column chromatography using DCM/EtOAc 95/5 as the eluent phase (*R*_f_ = 0.7). The pure compound was isolated as a yellow, viscous liquid (6.62 g) in 78% yield.

^1^H NMR (600 MHz, CDCl_3_) δ (ppm): 5.13–5.08 (m, 2H), 4.91–4.89 (t, *J* = 5.2 Hz, 1H), 4.81–4.71 (m, 4H), 4.55–4.54 (dt, *J* = 4.9, 1.0 Hz, 1H), 4.11–4.00 (m, 2H), 3.95–3.88 (m, 2H), 2.55–2.53 (td, *J* = 2.4, 1.5 Hz, 2H).

^13^C NMR (125 MHz, CDCl_3_) δ (ppm) = 153.9, 153.6, 85.8, 81.6, 80.9, 77.2, 76.1, 75.9, 73.2, 70.6, 55.7, 55.6.

HRMS: *m*/*z* [M + Na]^+^ calc. for [C_14_H_14_O_8_Na]^+^: 333.0581; found: 333.0606.

#### 2.2.3 Synthesis of Isomannide Dipropargyl Carbonate—ImDPC

In a 50 mL two-neck round-bottom flask equipped with a Dean–Stark apparatus, a water condenser, and a magnetic stir bar, isomannide (1.00 g, 6.80 mmol, 1.0 molar equiv) was dissolved in dipropargyl carbonate (9.40 g, 68.40 mmol, 10.0 molar equiv) in the presence of TBD (9.50 mg, 0.068 mmol, 0.01 mol eq.). The reaction mixture was heated at 100 °C for 2 h under magnetic stirring and nitrogen flow. Afterward, the hot reaction mixture was filtered through a silica pad on a Gooch filter under vacuum, and the silica pad was washed with EtOAc (20.0 mL × 3) to maximize product recovery. All the liquid was collected in a round-bottom flask and cooled down to room temperature. Ethyl acetate was removed by rotary evaporation under vacuum, and the dipropargyl carbonate excess was removed from the reaction mixture through a fractional distillation procedure under vacuum (*T*_(ext)_ = 70–75 °C, *p* = 30 mbar) as a transparent liquid and reused in subsequent trials. The product was obtained as pure via silica column chromatography (DCM/EtOAc 99/1) as a yellow, viscous liquid (1.26 g) in 60% yield.

^1^H NMR (600 MHz, CDCl_3_) δ (ppm) = 5.06–5.01 (tdd, *J* = 6.6 Hz, 2H), 4.81–4.70 (m, 6H), 4.09–4.05 (dd, *J* = 9.7,Hz, 2H), 3.92–3.88 (dd, *J* = 9.7 Hz, 2H), 2.54–2.53 (t, *J* = 2.5 Hz, 2H).

^13^C NMR (125 MHz, CDCl_3_) δ (ppm): 153.9, 80.2, 77.2, 76.6, 75.9, 70.3, 55.6.

HRMS: *m*/*z* [M + Na]^+^ calc. for [C_14_H_14_O_8_Na]^+^: 333.0581; found: 333.0611.

#### 2.2.4 Synthesis of Isosoidide Dipropargyl Carbonate—IiDPC

In a 50 mL two-neck round-bottom flask equipped with a Dean–Strak apparatus, a water condenser, and a magnetic stir bar, isoidide (1.00 g, 6.80 mmol, 1.0 mol equiv) was dissolved in dipropargyl carbonate (9.40 g, 68.40 mmol, 10.0 mol equiv) in the presence of TBD (9.50 mg, 0.068 mmol, 0.01 mol eq.). The reaction mixture was heated at 100 °C for 2 h under magnetic stirring and nitrogen flow. Afterward, the hot reaction mixture was filtered through a silica pad on a Gooch filter under vacuum, and the silica pad was washed with EtOAc (20.0 mL × 3) to maximize product recovery. All the liquid was collected in a round-bottom flask and cooled down to room temperature. Ethyl acetate was removed by rotary evaporation under vacuum, and the dipropargyl carbonate excess was removed from the reaction mixture through a fractional distillation procedure under vacuum (*T*_(ext)_ = 70–75 °C, *p* = 30 mbar) as a transparent liquid and reused in subsequent trials. The pure compound was isolated as a yellow, viscous liquid (2.08 g) in 98% yield without any chromatographic purification.

^1^H NMR (600 MHz, CDCl_3_) δ (ppm) = 5.14 (m, 2H), 4.75–4.72 (m, 6H), 4.04–3.95 (qd, *J* = 2.4 Hz, 4H), 2.56–2.54 (t, *J* = 2.5 Hz, 2H).

^13^C NMR (125 MHz, CDCl_3_) δ (ppm): 152.5, 84.1, 80.1, 75.5, 75.1, 71.3, 54.7.

HRMS: *m*/*z* [M + Na]^+^ calc. for [C_14_H_14_O_8_Na]^+^: 333.0581; found: 333.0606.

#### 2.2.5 Synthesis of Isosorbide Diallylcarbonate—IsDAllC

Diallylcarbonate was prepared according to the previously published procedure.^42^ In a 100 mL two-neck round-bottom flask equipped with a Dean–Stark apparatus, a water condenser, and a magnetic stir bar, isosorbide (2.00 g, 13.70 mmol, 1.0 molar equiv) was dissolved in diallyl carbonate (19.50 g, 0.13 mol, 10.0 molar equiv) in the presence of TBD (0.02 g, 0.13 mmol, 0.01 mol eq.). The reaction mixture was heated at 100 °C for 2 h under magnetic stirring and a nitrogen flow. Afterward, the hot reaction mixture was filtered through a silica pad on a Gooch filter under vacuum, and the silica pad was washed with EtOAc (20.0 mL × 3). All the liquid was collected in a round-bottom flask and cooled down to room temperature. Ethyl acetate was removed by rotary evaporation under vacuum, and the diallyl carbonate excess was removed from the reaction mixture through a fractional distillation procedure under vacuum (*T*_(ext)_ = 60–65 °C, *p* = 30 mbar) as a transparent liquid and reused in subsequent trials. The product was obtained as pure via silica column chromatography using DCM/EtOAc 95/5 as the eluent phase (*R*_f_ = 0.8). The pure compound was isolated as a yellow, viscous liquid (1.51 g) in 35% yield.

^1^H NMR (600 MHz, CDCl_3_) δ (ppm) = 5.93 (ddq, *J* = 17.3, 10.4, 5.8 Hz, 2H), 5.38 (dd, *J* = 2.7, 1.4 Hz, 1H), 5.35 (dd, *J* = 2.8, 1.4 Hz, 1H), 5.28 (ddd, *J* = 10.5, 5.4, 1.2 Hz, 2H), 5.11–5.06 (m, 2H), 4.89 (t, *J* = 5.1 Hz, 1H), 4.64 (ddt, *J* = 12.6, 5.8, 1.3 Hz, 4H), 4.54 (dt, *J* = 4.8, 1.0 Hz, 1H), 4.10–4.06 (m, 1H), 4.02 (dd, *J* = 11.0, 3.5 Hz, 1H), 3.91 (dd, *J* = 5.2, 4.3 Hz, 2H).

^13^C NMR (151 MHz, CDCl_3_) δ (ppm): 154.48, 154.15, 131.42, 131.28, 119.56, 119.22, 86.01, 81.37, 81.04, 76.92, 73.40, 70.64, 69.01, 68.97.

HRMS: *m*/*z* [M + Na]^+^ calc. for [C_14_H_14_O_8_Na]^+^: 337.0894; found: 337.1039.

### 2.3 Formulation Preparation

The TY formulations were prepared at a fixed stoichiometric ratio of 1:1 between the alkyne and the thiol reaction functional groups, considering each alkyne group as difunctional, resulting in an overall functionality of four reactive sites per monomer. TMPMP was used as the thiol component in all formulations. The cyclic-sugar-based alkyne monomers and TMPMP were weighed according to their functional group equivalence and mixed at room temperature until a homogeneous resin was obtained.

In a typical formulation preparation, IsDPC (3.00 g, 9.67 mmol) and TMPMP (5.10 g, 12.89 mmol) were mixed with a spatula for 3 min in a dark amber glass vial. BAPO was then added as a photoinitiator at 1 phr (parts per hundred resin; 0.081 g), as determined from the preliminary design of experiments (DoE) optimization (see Figure S1; Supporting Information). The mixture was sonicated for 10 min to ensure complete dissolution of the photoinitiator and stored in amber vials until use for further characterization.

#### 2.3.1 Postfunctionalization of the Cross-Linked Samples with PEGMA

The surface postfunctionalization of the cross-linked samples was carried out by submerging the cured specimens of alkyne-based materials in a PEGMA solution containing 1 phr BAPO. The solution had been previously sonicated for 10 min to ensure complete dissolution of the photoinitiator. The samples were then irradiated for 1 min under UV light at an intensity of 110 mW cm^–2^ to promote surface grafting.

### 2.4 Characterization

#### 2.4.1 Nuclear Magnetic Resonance and High-Resolution Mass Spectroscopy Analyses

NMR spectra were acquired through Bruker 400 and 600 MHz spectrometers in CDCl_3_. High-resolution mass spectra were recorded through a Bruker compact QTO, acquired in full scan positive polarity with a mass resolution of *R* = 30,000. The instrument calibration was conducted using a sodium formate cluster solution, and the data have been elaborated in HPC modality. The acquisition has been conducted in full scan mode in the interval between 50 and 500 *m*/*z* with a 4 l/min dry gas flow at 180 °C. The ionic formula of each compound has been calculated through the Smart Formula program, present inside the Bruker software using 4 mDa as mass confidence and considering the isotope pattern ratio.

#### 2.4.2 Fourier Transform Infrared Spectroscopy—FT-IR

The cross-linking reaction was followed by monitoring the decrease in characteristic IR absorbances of functional groups under illumination. For TY systems, one can follow the decay of the alkyne C≡C–H stretch (around 2100–2150 cm^–1^) and the thiol S–H stretch (∼2550 cm^–1^). By measuring these absorbances over time during UV exposure, conversion–time curves are obtained. Real-time FTIR has revealed the two-stage nature of TY cure (often a rapid initial consumption of alkyne followed by a secondary increase in vinyl sulfide conversion).

The Thermo Scientific Nicolet iS 50 Spectrometer was used to monitor the photocuring process in transmission mode. Then, the Omnic Thermo Fischer Scientific Software was used to handle the spectra afterward.

In transmission mode, a film of 12 μm of the liquid formulation was applied on a window made in SiC (silicon carbide), and scanned under a nitrogen atmosphere; then, the film was UV irradiated with increments of 5 s, and a spectrum was recorded. The UV-light source was around 110 mW/cm^2^, centered at 365 nm.

The wavenumber spectra recorded were between 400 and 4000 cm^–1^ with a resolution of 4 cm^–1^. The disappearance of alkyne (≡C–H) ∼ 3300 and 2130 cm^–1^ peaks and the thiol (S–H) group at 2250 cm^–1^ were monitored to calculate the conversion with the following relations:

$$\mathrm{Conversion}\;(\%)=\left(\frac{{\left(\frac{A_{1}}{A_{\mathrm{ref}}}\right)}_{t=0}-{\left(\frac{A_{1}}{A_{\mathrm{ref}}}\right)}_{t}}{{\left(\frac{A_{1}}{A_{\mathrm{ref}}}\right)}_{t=0}}\right)\times 100$$ 1

where *A*_ref_ is the peak taken as reference at 1746 cm^–1^, which does not change with the proceeding of the cross-linking reaction. *A*_1_ is the peak under investigation.

#### 2.4.3 Photo-Dynamic Scanning Calorimetry—Photo-DSC

The photo-DSC method was utilized to investigate the process of photocuring. Analysis was carried out using a Mettler TOLEDO DSC-1 with a Gas Controller GC100. A mercury lamp, Hamamatsu LIGHTINGCURE LC8, with an optical fiber was used to directly irradiate the samples with UV light at 365 nm with an intensity of approximately 110 mW/cm^2^, while an empty pan served as a reference. The analysis was conducted at RT in a nitrogen atmosphere with a flow rate of 40 mL/min.

The samples were irradiated for two cycles. The second cycle was performed to confirm complete curing and establish a baseline; then, the second curve was subtracted from the first. The energy resulting from the various samples, as the integration of the curve, was then compared to the pristine reagents. The time at which the peak occurs gives an estimation of the reactivity of the system.

#### 2.4.4 Dynamic Mechanical Thermal Analysis—DMTA

A Triton Technology instrument was used for the thermo-mechanical analysis in the temperature sweep mode. The temperature ramp was set at a rate of 3 °C/min beginning from *r* −30 to 180 °C. The applied mechanical stress frequency was 1 Hz, and the strain rate was 0.1% using the tensile method. The value of the glass transition was determined by the peak of the tanδ curve.^41^ The samples tested were UV-cured in silicon molds.

#### 2.4.5 Curing Behavior and Printing Parameters

The printing parameters were optimized using an Asiga MAX X DLP printer (Asiga, Erfurt, Germany) equipped with a 385 nm light source operating at 19 mW cm^–2^. To define the working conditions, the isosorbide-based formulation was examined by constructing working curves that related the curing depth (*C*_d_) to the exposure energy. For each test, resin films were irradiated for different time intervals, and the thickness of the polymerized layers was measured with a MITUTOYO IP65 digital micrometer (Mitutoyo Europe GmbH).

The measurements were used to calculate *C*_d_ and determine the critical exposure energy (*E*_c_) from the linear regression of eq 2, where *D*_p_ represents the penetration depth of light into the resin and *E*_c_ is the critical exposure energy required to initiate resin polymerization.^42,43^

$$C_{d}=D_{p}\ln\left(\frac{E_{\max}}{E_{c}}\right)$$ 2

Printing of the isosorbide-based resin was then carried out by using an exposure time of 20 s per 25 μm layer. The printed objects were washed in isopropanol for 5 min in an ultrasonic bath, air-dried, and postcured for 30 min under 405 nm irradiation to achieve full cross-linking.

## 3 Results and Discussion

### 3.1 Synthesis of the Dipropargyl Carbonate Monomers

The synthesis of IsDPC was initially investigated via transcarbonylation of dimethoxycarbonyl isosorbide (DCI) with propargyl alcohol (Scheme 2, pathway A) promoted by a catalytic amount of 1,5,7-triazabicyclo[4.4.0]­dec-5-ene (TBD). DCI is available in large amounts in our laboratory from previous studies.^40,41^ Unfortunately, this procedure led to the formation of propargyl methyl carbonate and isosorbide as the main products.

In a second approach, the synthesis of IsDPC was pursued via alkoxycarbonylation of isosorbide using dipropargyl carbonate (Scheme 2, pathway B). As this method required dipropargyl carbonate, its preparation was also investigated here for the first time.

The synthesis of the novel dialkyne carbonate was carried out by modifying an already published procedure (Scheme 3).^42^ This methodology is based on a double transcarbonylation of dimethyl carbonate (DMC) with propargyl alcohol in the presence of TBD as a homogeneous basic catalyst. In addition to the target product, the reaction may also produce methyl propargyl carbonate as a side product.

**Scheme 3:**
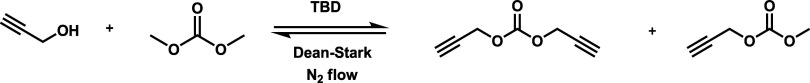
Synthesis of Dipropargyl Carbonate

In a typical reaction, DMC (1.0 mol equiv) and an excess of propargyl alcohol (5.0 mol equiv) were mixed in the presence of TBD (0.01 mol equiv) as a base. A Dean–Stark apparatus and a nitrogen flow were employed to drive the equilibrium forward by removing methanol formed as a side product. Several optimization tests were carried out in order to obtain dipropargyl carbonate with the highest yield, by varying different parameters (Table S1; Supporting Information).

The optimal conditions were obtained when the reaction was carried out for a total of 8 h—4 h with a condenser and the remaining time with a Dean–Stark apparatus and N_2_ flow at 95 °C—using 0.02 mol equiv of TBD. In these conditions dipropargyl carbonate was isolated in moderate yield most probably because of its instability at high temperatures. In fact, when distillation of the product was attempted, the mixture turned black, and a sticky residue formed at the bottom of the flask. The only way to avoid this issue was to first distill the residual propargyl alcohol at low temperature (50 °C) and then filter the resulting mixture through silica to remove residual TBD, allowing the pure product to be recovered as an orange oil. It should be mentioned that despite the moderate yield, this procedure can be easily scaled up and the excess of propargyl alcohol recovered and reused.

Green metrics were also calculated for this procedure (see the SI). The atom economy of the reaction was approximately 70%, which is acceptable considering that two mol of methanol are released as leaving groups for each mole of product formed. The E-factor was calculated to be 11.6 (Table S2, SI), with the major contributions arising from (i) the E-kernel associated with byproducts (methanol) and unreacted starting material (propargyl alcohol), and (ii) E-purif due to the use of ethyl acetate during product purification. Although this value is already within an acceptable range, it could be further improved by developing more efficient reaction conditions.

The synthesis of isosorbide dipropargyl carbonate (IsDPC) was then carried out via a double alkoxycarbonylation pathway, as described in Scheme 2 (pathway B).

In a typical reaction, isosorbide and an excess of dipropargyl carbonate were added to a two-neck round-bottom flask in the presence of TBD as a catalyst. Also in this case, a Dean–Stark apparatus and a nitrogen flow were employed to facilitate the removal of methanol forming as a side product, ultimately driving the reaction forward. Optimization tests were carried out, varying the amount of catalyst, the reaction temperature, and time. Results collected are reported in Table 1.

**Table 1:** Optimization Reactions for the Synthesis of IsDPC^a^

#	**dipropargyl carb. (mol. eq)**	**TBD (mol. eq)**	***T* (°C)**	***t* (h)**	**yield IsDPC (%)^b^**
1	10	0.01	120	2	53
2	10	0.01	100	2	78
3	10	0.01	80	2	60
4	5	0.01	100	2	47
5	10	0.02	100	2	42

a Reaction conditions: isosorbide (1.0 mol eq), propargyl carbonate, and TBD were mixed in a 100 mL two-neck round-bottom flask in the presence of a Dean–Stark apparatus under nitrogen flow; isosorbide conversion was always quantitative.

b Isolated yield.

In particular, the temperature required a fine-tuning. The best result was achieved when the reaction was conducted at 100 °C (#2, 1) as IsDPC was isolated in very good yield, i.e., 78%. When trials were conducted at either higher (no. 1; Table 1) or lower temperature (no. 3; Table 1), the yield decreased. Furthermore, a large excess of dipropargyl carbonate (10.0 mol eq) seemed to be necessary to maintain high IsDPC yields. In fact, using 5.0 mol equiv of dipropargyl carbonate resulted in a lower product recovery (#4, Table 1). Similarly, doubling the amount of TBD did not improve the yield of IsDPC (#5, Table 1 **).**

In all trials reported in Table 1, IsDPC was isolated by column chromatography after distillation of excess dipropargyl carbonate, which was subsequently reused in other experiments. In contrast, despite our best effort the monopropargyl carbonate derivative of isosorbide (IsMPC) was never isolated, most probably because it degrades rapidly upon formation.

In conclusion, the optimal conditions were found to be 10.0 mol eq of propargyl carbonate and 0.01 mol eq of TBD at 100 °C for 2 h with a Dean–Stark apparatus and a nitrogen flow.

Green metrics for the preparation of IsDPC were then evaluated (see SI). The E-factor for this procedure (Table S2) was calculated to be 14, with the major contributions arising from (i) the E-reaction solvent due to the excess of propargyl carbonate and (ii) E-purif associated with the use of ethyl acetate. It should be noted that this E-factor value is underestimated, as it does not include the mass of materials and solvents used for column chromatography. The latter was necessary to ensure the high purity of the monomer required for efficient thiol–yne polymerization.

The optimized conditions found for IsDPC (no. 2, Table 1) were then applied for the synthesis of two additional alkyneyl derived using as substrates the epimers of isosorbide, i.e., isomannide and isoidide (Figure 2, Table 2).

**Figure 2:**
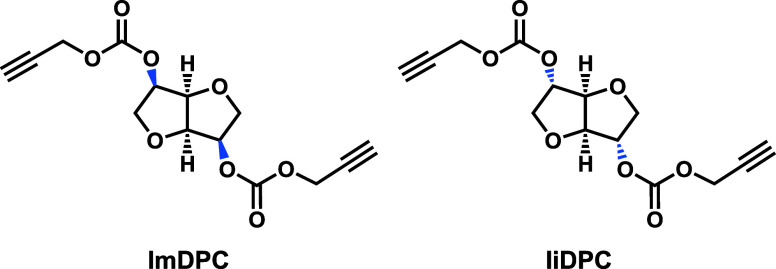
Chemical structures of ImDPC and IiDPC.

**Table 2:** Optimization Reactions for the Synthesis of ImDPC and IiDPC^a^

cyclic sugar	**dipropargyl carb. (mol eq)**	**TBD (mol eq)**	***T* (°C)**	***t* (h)**	**conv. (%)**	**yield (%)^b^**
isomannide	10	0.01	100	2	100	**ImDPC** 60
isoidide	10	0.01	100	2	100	**IiDPC** 98^c^

a Reaction conditions: The selected sugar (1.0 g, 1.0 mol eq), propargyl carbonate (9.40 g, 10.0 mol eq), and TBD (9.50 mg, 0.01 mol eq) were reacted in the presence of a Dean–Stark apparatus under a nitrogen flow.

b Isolated yield via column chromatography.

c Product isolated without any purification.

The results obtained confirm the different reactivity between isosorbide and its epimers, due to the different orientation of the hydroxyl groups. In particular, the reactions involving isomannide as a substrate led to a lower yield (60%, Table 2) compared to isosorbide because of the two *endo* hydroxyl groups, which are less reactive due to their sterically hindered conformation. Conversely, when isoidide was used, the alkoxycarbonylation reaction proceeded quantitatively (98% yield, Table 2) due to the higher reactivity of the *exo* hydroxyl groups. Due to the high efficiency of this latest reaction, IiDPC was isolated as pure without any chromatographic purifications. It is interesting to mention that isosorbide exhibits an intermediate reactivity between its two epimers, with an isolated yield of 78%. The results obtained for these three cyclic sugars are perfectly consistent with our previous studies on their reactivity.^35,48^

### 3.2 Thiol–Yne Polymerization: Investigation of the UV-Curing Process

Design of experiment (DoE) is essential in the early stages of formulation development, as it allows a systematic and statistically robust identification of the parameters that most significantly influence the curing process. This approach reduces the number of required experiments to map the polymerization behavior and provides quantitative insight into the optimal conditions for efficient and reproducible curing. DoE was carried out using photo-DSC by varying the light intensity between 40 and 200 mW cm^–2^ and the photoinitiator content between 0.5 and 2 phr on the TMPMP–IsDPC, TMPMP–ImDPC, and TMPMP–IiDPC formulations, while maintaining a fixed 1:1 stoichiometric ratio between the alkyne and thiol functional groups (Figure S1; Supporting Information). To fully dissolve the photoinitiator, the formulations underwent sonication at room temperature for 10 min. Shielded vials were used to avoid exposure to light.

The three formulations exhibited comparable trends, and based on these results, the photoinitiator concentration was set at 1 phr and the light intensity at 110 mW cm^–2^ for all subsequent investigations. In Table 3 and Figure 3 are reported all of the investigated formulations.

**Figure 3:**
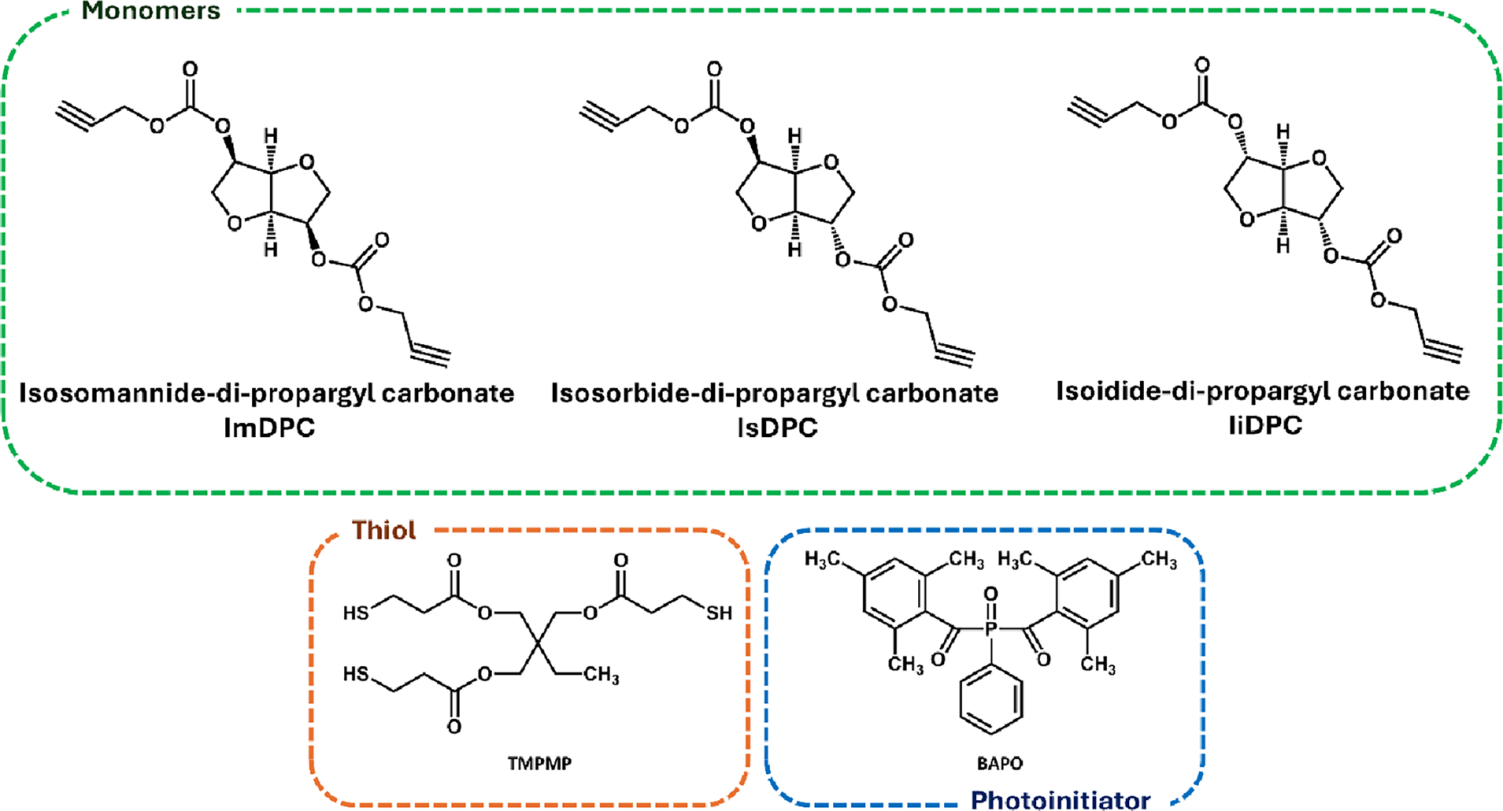
Chemical structures of the monomers, thiol, and photoinitiator employed in the formulations.

**Table 3:** Summary of the Formulations Studied for TY Polymerization^a^

**alkyne**	**thiol**	**BAPO (1 phr)**	**code**
IsDPC	TMPMP	1	**TMPMP—IsDPC**
ImDPC	TMPMP	1	**TMPMP—ImDPC**
IiDPC	TMPMP	1	**TMPMP—IiDPC**

a Reaction conditions: The TY formulations were prepared at a fixed stoichiometric ratio of 1:1 between the alkyne and the thiol reaction functional groups. The cyclic sugar-based alkyne monomers and TMPMP were weighed according to their functional group equivalence and mixed at room temperature until a homogeneous resin was obtained.

IsDPC, ImDPC, and IiDPC TY-based formulations were investigated under UV irradiation via FT-IR analysis. The peaks at 3300 cm^–1^ (≡C–H) and 2130 cm^–1^ were monitored to determine alkyne conversion while the area of the S–H band, around 2550 cm^–1^, was followed to investigate the thiol group conversion under irradiation. The conversion curves as a function of the irradiation time for each formulation are reported in Figure 4.

**Figure 4:**
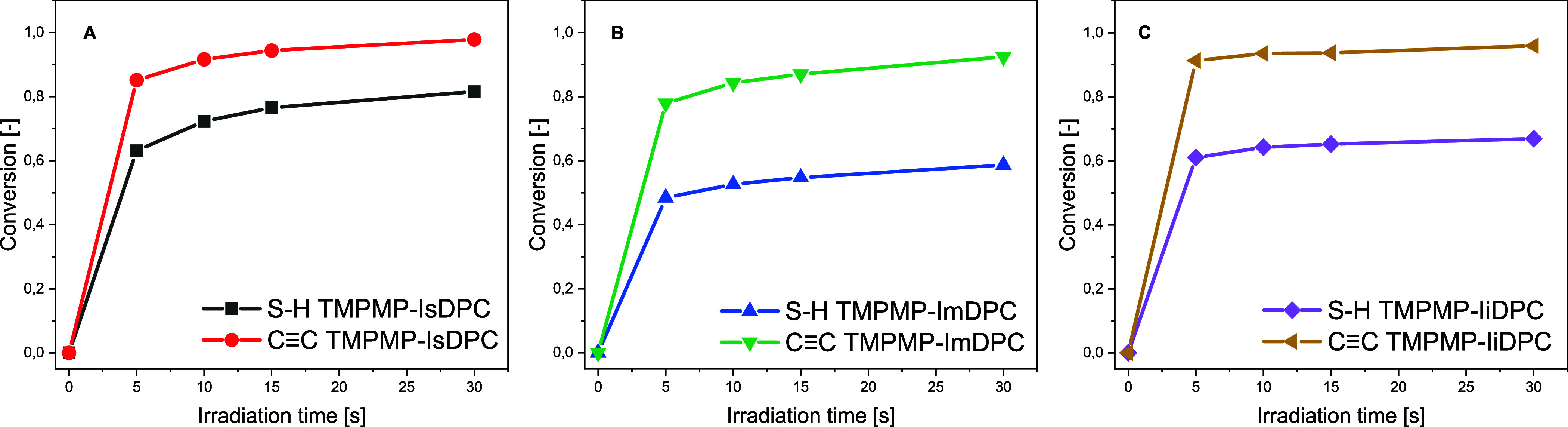
Conversion trend for the TMPMP-IsDPC (A), TMPMP-ImDPC (B), and TMPMP-IiDPC (C) formulations.

The curves clearly show a high reactivity of the alkyne groups toward the TY reaction, where for all investigated isosorbide isomers an almost complete conversion (above 95%) is achieved after 30 s of irradiation.

In contrast, thiol conversion varies among the three isomers, and it is possible to observe a thiol conversion of about 58% for the ImDPC-based system, 66% for the IiDPC-derived formulation, and up to 81% for the IsDPC monomer. This variation can be attributed to the radical-mediated homopolymerization of the alkyne groups and the vinyl sulfide intermediates,^1^ respectively, reducing the availability of reactive sites for thiol addition.^7,49,50^

To verify this hypothesis, the allyl analogue of the isosorbide-based alkyne monomer (IsDAllC) was synthesized and reacted with TMPMP under identical conditions. The photopolymerization process was monitored in real time. As shown in Figure 5, the reaction displays the characteristic behavior of a thiol–ene click mechanism, where the disappearance of both the C=C and S–H absorption bands occurs simultaneously, reaching nearly complete conversion within a few seconds of irradiation. The absorption bands at 810 and 2550 cm^–1^, corresponding to the alkene and thiol stretching vibrations, respectively, were monitored to calculate the functional group conversion according to eq 1.

**Figure 5:**
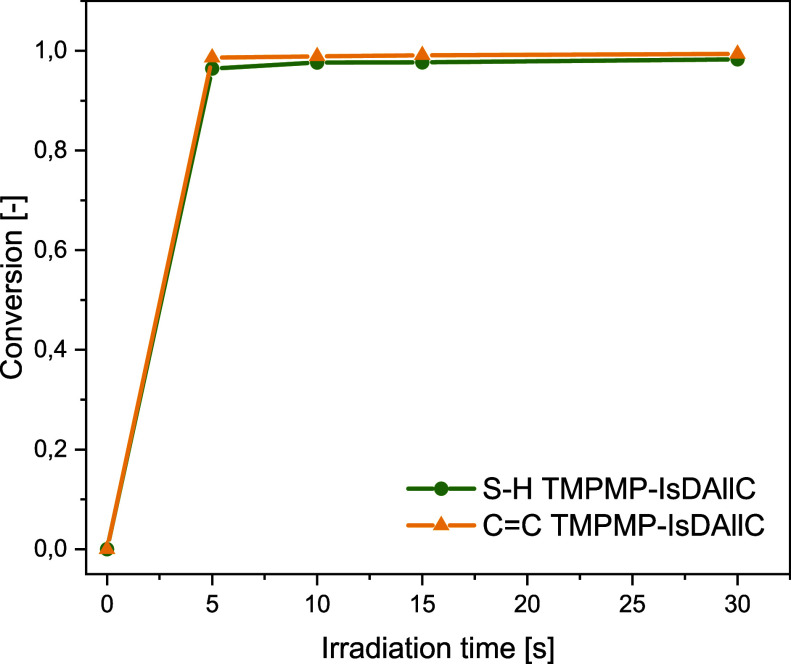
Photopolymerization kinetics and conversion of the TMPMP-IsDAllC thiol–ene formulation.

It should be noted that in the investigated different TY formulations, the residual thiol groups present on the surface after UV curing can be exploited for postfunctionalization with poly­(ethylene glycol) methyl ether methacrylate (PEGMA) (see Section 3.3).

Photo-DSC analysis was also conducted to validate the conversion trends observed by FTIR spectroscopy of the three TY formulations. As shown in Figure 6, the TY isosorbide-based formulation exhibits the highest total heat release during the cross-linking process (363 J g^–1^), followed by the isomannide-based system (352 J g^–1^) and the isoidide-based formulation (350 J g^–1^), which showed similar heat release. The photo-DSC data are quite in agreement with FT-IR analysis, showing the higher alkyne triple bond conversion of isosorbide toward TY reaction, compared with the isomannide and isoidide epimers.

**Figure 6:**
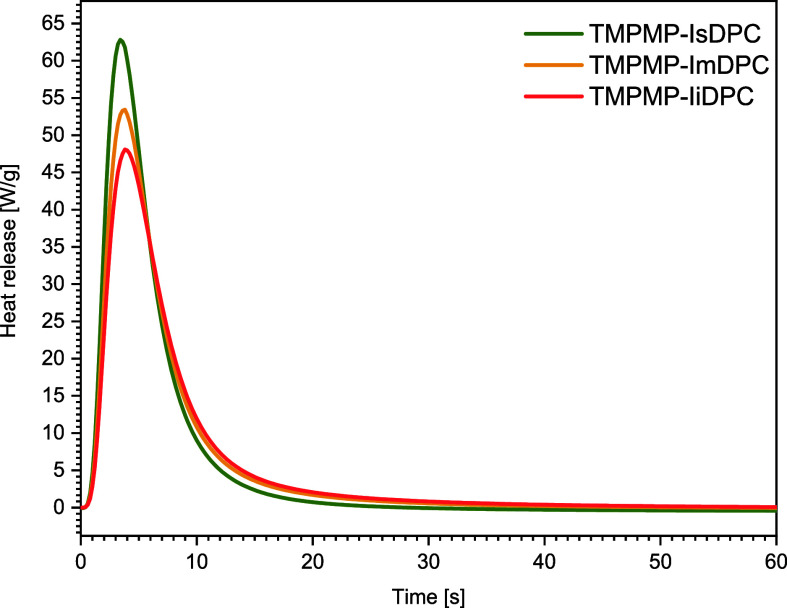
Heat release profiles obtained from photo-DSC for the different TY formulations.

### 3.3 Thermo-Mechanical Analysis of the Thiol–Yne Formulations

The three UV-cured formulations, TMPMP-IsDPC, TMPMP-ImDPC, and TMPMP-IiDPC, were characterized by DSC and DMTA analysis. The thermal properties of UV-cured films were measured by DSC analysis, showing a slight difference in the *T*_g_ for the three formulations. The data are collected in Table 4 showing that all the cross-linked formulations are very flexible with low *T*_g_ although the isosorbide-based formulation showed a higher *T*_g_ value of 1 °C, compared with the value of ca −6 °C for the isomannide- and −8 °C for the isoidide-based formulations. These data are in agreement with the overall thiol-functional group conversion that followed the same trend and therefore can be attributed to a decrease of cross-linking density when using isomannide or isoidide with respect to the isosorbide alkyne monomer.

**Table 4:** Heat Flow Values and DSC *T*_g_ Values for Different TY Formulations

**formulations**	**FTIR conversions** C≡C/S–H	**heat release** (J/g)	***T***_**g**_ **(°C)** DSC	***T***_**g**_ **(°C)** DMTA
**TMPMP-IsDPC**	0.98/0.82	363	1	48
**TMPMP-ImDPC**	0.92/0.60	352	–6	33
**TMPMP-IiDPC**	0.96/0.67	350	–8	31

The viscoelastic properties of the cured materials were evaluated by DMTA. In Figure 7B, the storage modulus (*E*’), loss modulus (*E*″), and the tanδ curves are reported for the 3 different formulations. *T*_g_ is given as the maximum of tan δ curves. The data reported in Figure 7 clearly show that the viscoelastic behavior of isomannide- and isoidide-based formulations are very similar, with the same trend in E’ drop and a very close *T*_g_ of 33 and 31 °C, respectively .

**Figure 7:**
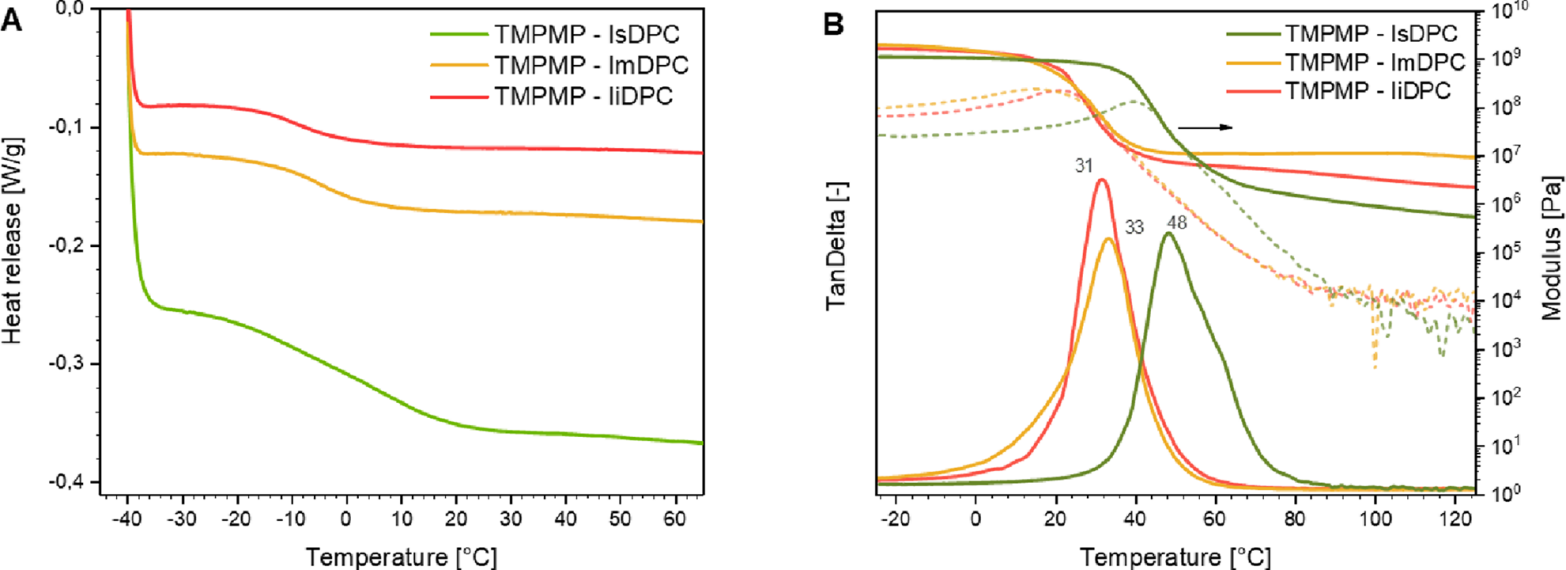
(A) DSC curves for the different TY formulations and (B) DMTA curves (tan δ vs temperature) for all three cross-linked TY materials.

On the other hand, the photocured isosorbide-based formulation showed a decrease of *E*′ modulus to higher temperature with a shift of the maximum of tanδ peak to 48 °C. The higher *T*_g_ values determined by DMTA analysis, with respect to the ones of the DSC measurements, are due to a very well-known resonance effect and it is clearly reported in literature.^51,52^ In any case, the trend is confirming the DSC data, showing the higher thermo-mechanical performance for the isosorbide-based formulation.

### 3.4 Surface Postfunctionalization of the Thiol–Yne Formulations

Since the S–H functional groups of the three TY formulations herein investigated were not completely consumed during the photocuring process, the residual thiol groups on the surface can be utilized for a postfunctionalization with poly­(ethylene glycol) methyl ether methacrylate (PEGMA, M^n^ ≈ 360) to enhance surface hydrophilicity. For this purpose, 1 phr of BAPO photoinitiator was dissolved into the PEGMA, which was then applied onto the isosorbide-based cross-linked materials by dipping and irradiated to promote the thiol–ene coupling reaction. The residual thiol-groups were evidenced by ATR-FTIR analysis and their consumption upon postfunctionalization was monitored and reported in Figure 8. The consumption of the SH groups evidenced their exploitation during the thio-methacrylate addition reaction promoted by UV light in the presence of the radical photoinitiator (Figure 8A).

**Figure 8:**
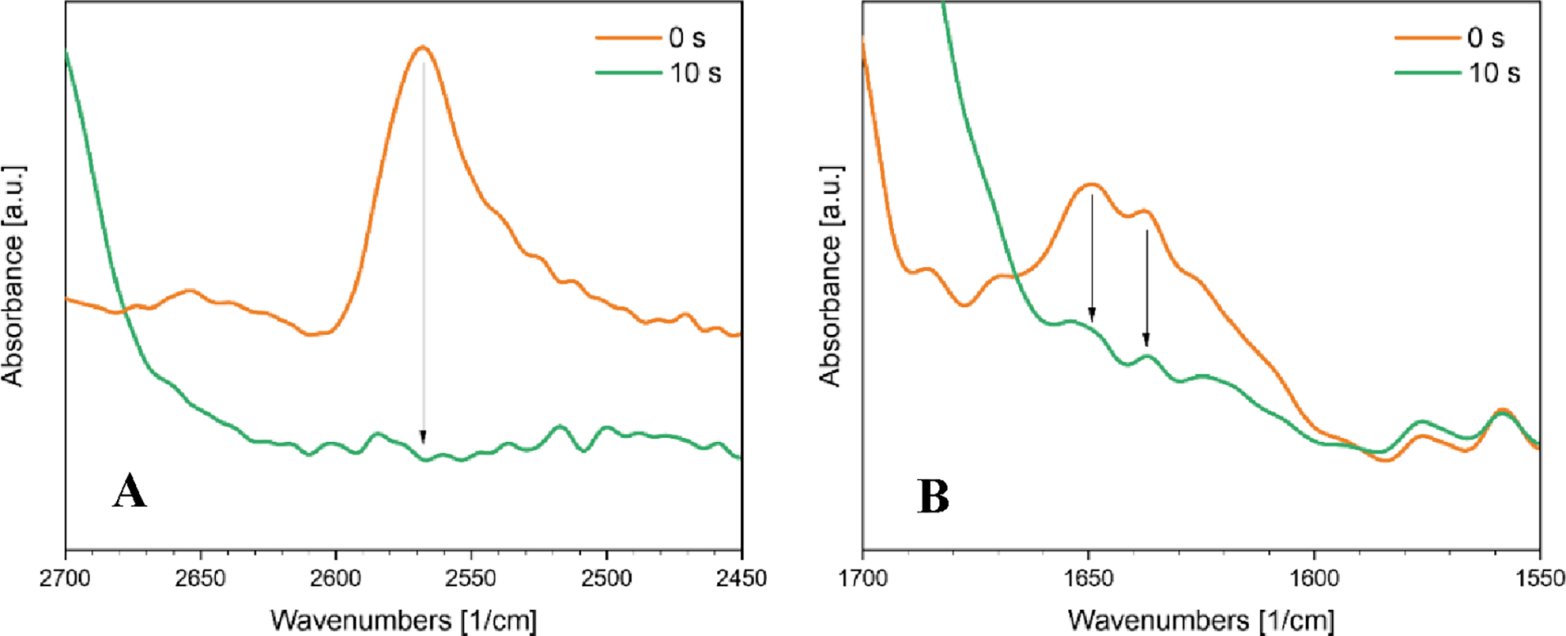
ATR-FTIR spectra showing (A) the disappearance of methacrylate-related peaks after PEGMA surface functionalization and (B) the disappearance of the S–H stretching band.

Following the PEGMA functionalization, the characteristic peaks corresponding to both the thiol and methacrylate groups nearly disappeared in the ATR-FTIR spectrum (Figure 8B), confirming surface modification. As shown in Figure 9, the static water contact angle decreased dramatically from 86° for the UV-cured TY-based formulation (Figure 9A) to 23° after postfunctionalization with the hydrophilic PEGMA (Figure 9B). A complete wetting was achieved within a few seconds, indicating a significant enhancement in surface wettability and demonstrating the feasibility of exploiting the residual thiol-groups unreacted on the surface of the UV-cured films.

**Figure 9:**
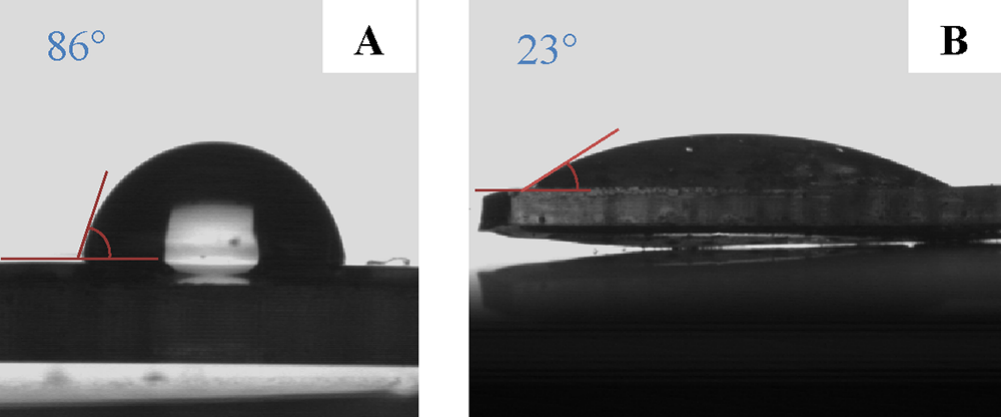
Water contact angle measurements (A) before and (B) after PEGMA surface functionalization.

### 3.5 3D-Printing of the Isosorbide-Based Formulation

The isosorbide-based formulation (3SH-IsDPC) was selected for sample fabrication using a DLP-based 3D printing process, and the photopolymerization behavior under layer-by-layer exposure was characterized through Jacobs working curve analysis; the cured depth follows eq 2. The working curve, which relates the cured layer thickness (*C*_d_) to the logarithm of the exposure energy (*E*), provides fundamental parameters describing the resin’s sensitivity and light attenuation (Figure 10).

**Figure 10:**
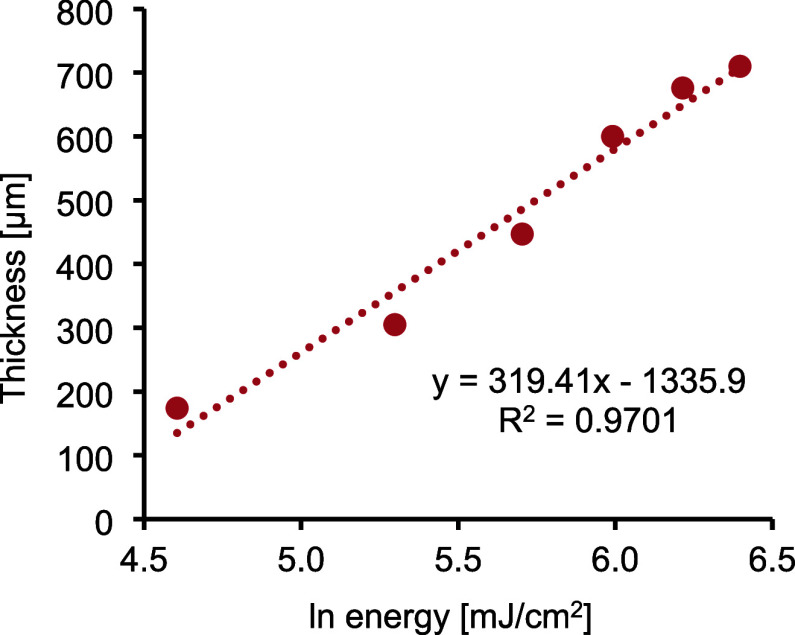
Working curve of the TMPMP-IsDPC formulation obtained from the DLP printing process.

The slope of the curve (*D*_p_ = 319.41 μm) defines the characteristic depth at which the irradiance falls to 1/e of its surface value and thus quantifies the light penetration through the material. A relatively large *D*_p_ value implies moderate optical attenuation and high resin transparency, consistent with the low light scattering typically observed in TY systems. This behavior facilitates accurate z-resolution control and effective interlayer adhesion during printing. The critical exposure energy (*E*_c_ = 2.05 mJ cm^–2^) derived from the curve represents the minimum energy density required to reach the gelation threshold.

The combination of *E*_c_ and *D*_p_ can be an indication of the printability of the formulation considering the exposure times, layer adhesion, and dimensional accuracy. The optimized exposure window was subsequently used to fabricate test structures with well-defined geometries, confirming the consistency of the curing response and validating the suitability of the TMPMP-IsDPC formulation for additive manufacturing.

The corresponding 3D printed samples obtained under the optimized exposure conditions are shown in Figure 11.

**Figure 11:**
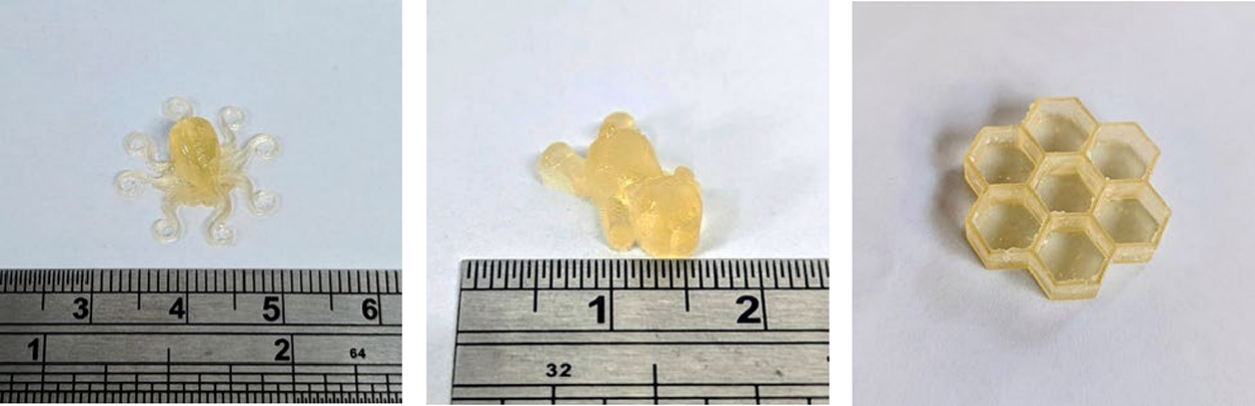
3D-printed specimens produced from the 3SH-IsDPC formulation under the optimized DLP printing conditions.

The printed structures exhibited good definition, confirming that the selected processing parameters ensured adequate curing depth and interlayer adhesion. Complex shapes were reproduced with good accuracy, demonstrating the formulation’s favorable balance between light penetration and spatial resolution. This result validates the suitability of the TMPMP–IsDPC resin for DLP printing.

## 4 Conclusions

Biobased TY systems demonstrated good potential as sustainable alternatives to traditional petroleum-derived photopolymers. Their step-growth radical mechanism enables rapid and homogeneous network formation with high conversion, limited oxygen inhibition, and tunable mechanical performance.

In particular, this work focuses on the synthesis of isosorbide, isomannide, and isoidide propargyl carbonate monomers in good yields via the alkoxycarbonylation reaction. These biobased dialkyne compounds were exploited in TY reactions using tris­(3-mercaptopropionate) as the thiol counterpart, following the curing process both by FT-IR analysis and photo-DSC.

Data collected showed that alkyne groups and the vinyl sulfide intermediates exhibit a certain tendency toward homopolymerization. Unlike the thiol–ene click reaction, partial homopolymerization of alkynes leads to faster consumption of C≡C bonds, thereby reducing the number of reactive sites available for thiol addition. Among the tested monomers, the isosorbide-based dialkyne showed the highest thiol consumption upon irradiation, while isomannide- and isoidide-based systems exhibited similar but lower thiol conversions. The unreacted thiol groups remaining in the UV-cured films can be exploited for further functionalization. As an example in the isosorbide-based formulation, additional thiol consumption was efficiently induced by UV irradiation in the presence of poly­(ethylene glycol) methacrylate (PEGMA). This secondary click reaction significantly modified the surface properties of the UV-cured films: the static water contact angle dropped sharply from 89° (for the UV-cured isosorbide-based TY formulation) to 23° after postfunctionalization with hydrophilic PEGMA.

The optimized isosorbide-based formulations developed in this study also exhibited excellent photocuring efficiency and 3D printability, confirming their suitability for DLP technologies and enabling the fabrication of high-resolution, complex 3D-printed structures.

Overall, this study highlights the versatility of TY chemistry as a robust platform for designing renewable, high-performance photoresins for advanced additive manufacturing applications.

## Supplementary Material





## Notes

Reaction conditions for the synthesis of dipropargyl carbonate, design of experiment performed using photo-DSC measuring the heat release, NMR spectra (^1^H and ^13^C spectra), and HRMS analysis of all the monomers used in this article (PDF)
Synthesis of dipropargyl carbonate and IsDPC and summary (XLS)
