# Supplementary material for: Thiol–Yne Photocurable Isosorbide-Derived Networks: Formulation and 3D Printing
Source: ACS Sustain Chem Eng. 2026 Feb 4;14(6):3258–70. doi: 10.1021/acssuschemeng.5c13600 (PMC12918243; doi:10.1021/acssuschemeng.5c13600)
Supplement: Supplementary file 1 [file sc5c13600_si_001.pdf]

<u>Supporting information</u>

Thiol-Yne Photocurable Isosorbide-derived Networks: Formulation and 3D Printing

Dumitru Moraru,[a,‡] Giacomo Trapasso,[b,‡] Davide Dalla Torre,[b] Thomas Griesser,[c] Fabio Aricò,[b,] Marco Sangermano[a,*]*

CONTENTS

1. Reactions conditions for the synthesis of dipropargyl carbonate. 3

2. Design of Experiment performed using photo-DSC measuring the heat release 3

3. Green Metrics Evaluation 4

3.1 Green metrics formulas 4

4. NMR Spectra and HRMS analysis 6

1. Reactions conditions for the synthesis of dipropargyl carbonate.

Table S1. Optimization reactions for the synthesis of dipropargyl carbonate.[a]

#	Propargyl alcohol (mol. eq.)	TBD (mol. eq.)	T (°C)	t (h)	Selectivity (%)		Yield (%)[b]
					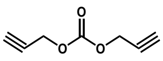	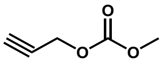	
1	5.0	0.01	95	8[c]	100	0	22
2	5.0	0.02	95	8[c]	85	15	32
3	5.0	0.04	95	8[c]	86	14	36
4	5.0	0.05	95	8[c]	93	7	20
5	5.0	0.02	95	4[d]	73	27	24
6	5.0	0.02	95	16[d]	31	69	5
7	8.0	0.02	95	8[c]	71	29	13
8	5.0	0.02	95	8	100	0	41
9	5.0	0.02	80	24[e]	91	6	18

[a] Reaction conditions: DMC (10.0 mL, 0.119 mol, 1.0 mol eq.), propargyl alcohol, and TBD were mixed in a 100 mL two-neck round-bottom flask and maintained at the set temperature for 4 hours in the presence of a condenser. Subsequently, a Dean–Stark apparatus with a nitrogen flow was connected for the remaining reaction time. [b] Isolated yield after distilling off the excess alcohol, filtering the remaining mixture through a vacuum silica pad and washing with EtOAc. [c] 4 hours with condenser followed by 2 hours with Dean–Stark under nitrogen flow, and finally another 2 hours with condenser. Procedure previously reported in the literature.[1] [d] Reaction performed using only the condenser without the Dean-Stark apparatus. [e] 4 hours with condenser and 16 hours with the Dean–Stark apparatus under nitrogen flow.

2. Design of Experiment performed using photo-DSC measuring the heat release

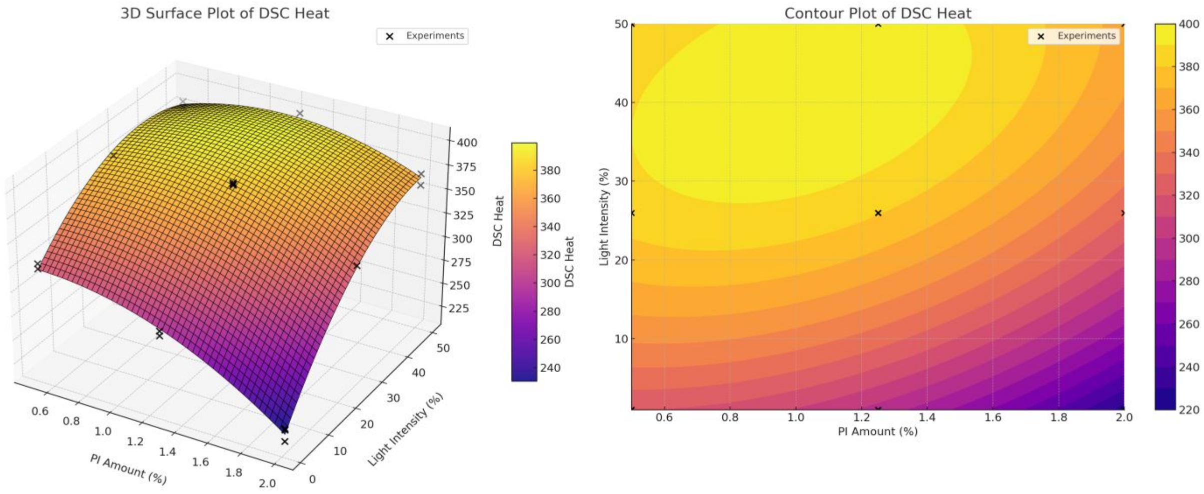

Figure S1. DoE surface plot (*left*) and the DoE plotted results (*right*).

[1] G. Trapasso, C. Salaris, M. Reich, E. Logunova, C. Salata, K. Kümmerer, A. Figoli and F. Aricò, *Sust. Chem. Pharm*, 2022, **26**, 100639.

3. Green Metrics Evaluation

With the aim of assessing the greenness of our two-step procedure for the synthesis of IsDPC, green metrics were evaluated. For each step, Atom Economy (AE), Process Mass Intensity (PMI), Reaction Mass Efficiency (RME), Material Recovery Parameter (MRP), and E-factor were calculated[2] and visually summarized using the Andraos radial pentagon.[3]

3.1 Green metrics formulas[4]

Metric	Abbreviation	Formula
Atom economy	AE	$\frac{\text{Molar mass of product}}{\text{Molar mass of all reactants}} \text{x100}$
Environmental factor	*E*-factor	$\frac{\text{Total mass of waste (kg)}}{\text{Mass of product (kg)}}$
Process mass intensity	PMI	$\frac{\text{Total mass used in the process (kg)}}{\text{Mass of product (kg)}}$
Reaction mass efficiency	RME	$\frac{\text{Mass of product (kg)}}{\text{Total mass of reactants (kg)}} \text{x100}$
Material recovery parameter	MRP	$\frac{\text{Total mass solvents + mass of catalyst recovered (kg)}}{\text{Total mass of solvents + mass of catalysts used (kg)}}$
Stoichiometric factor	SF	$1 + \frac{\text{Total mass of excess reagents (kg)}}{\text{Total mass of stoichiometric reagents (kg)}}$

Waste-related Green Metrics:

- E-kernel: Mass contribution to the total E-factor from reaction by-products, reaction side products, and unreacted starting materials;

- E-reaction solvent (E-rxn solv): Mass of reaction solvent necessary for the synthesis of the target product;

- E-catalyst (E-cat): Mass of the catalyst necessary for the synthesis of the target product;

- E-workup: Mass of the reagents used in the work-up procedures necessary to obtain the target product;

---

2 Andraos, J. and Hent, A. Simplified Application of Material Efficiency Green Metrics to Synthesis Plans: Pedagogical Case Studies Selected from *Organic Syntheses*. *J. Chem. Educ.* **2015**, *92* (11), 1820–1830.

3 Andraos, J. and Sayed, M. On the Use of "Green" Metrics in the Undergraduate Organic Chemistry Lecture and Lab To Assess the Mass Efficiency of Organic Reactions. *J. Chem. Educ.* **2007**, *84* (6), 1004

4 Fantozzi, N.; Volle, J.-N.; Porcheddu, A.; Virieux, D.; García, F.; Colacino, E. Green Metrics in Mechanochemistry. *Chem. Soc. Rev.* **2023**, *52* (19), 6680–6714.

- E-purification (E-purif): Mass of the reagents used in purification procedures necessary to obtain the pure target product.

Step 1: Dipropargyl carbonate synthesis

Metric	Value
AE	0.683
Rxn Yield	0.410
RME	0.079
MRP	0.282
1/SF	1.000

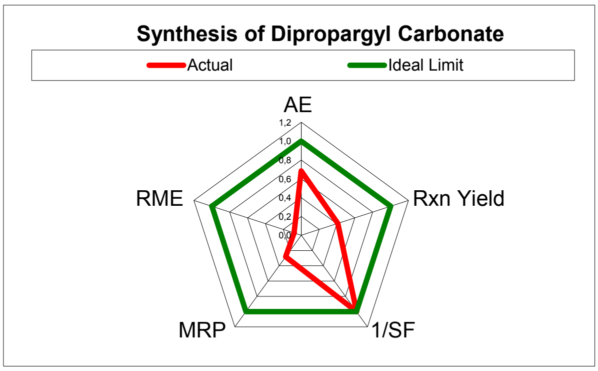

Step 2: Isosorbide dipropargyl carbonate

Metric	Value
AE	0.735
Rxn Yield	0.780
RME	0.065
MRP	0.114
1/SF	1.000

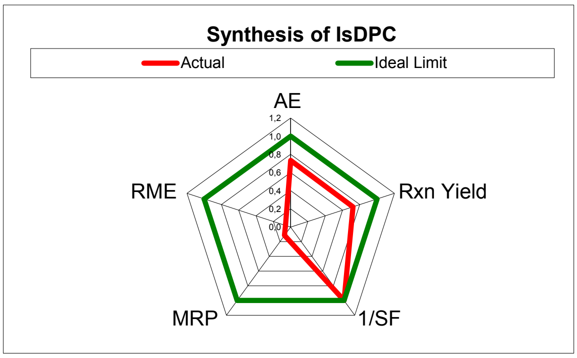

Table S2. Environmental assessment of two steps reaction synthesis of IsDPC

#	Method	Yield (%)	E-kernel	E-rxn sol.	E-cat	E-purif	E-factor
1	Step 1	41	2.54	0.33	0.05	8.72	11.64
2	Step 2	78	0.75	4.64	0.01	8.93	14.33

4. NMR Spectra and HRMS analysis

Figure S2: ^{1}H NMR of **dipropargyl carbonate** in $CDCl_3$

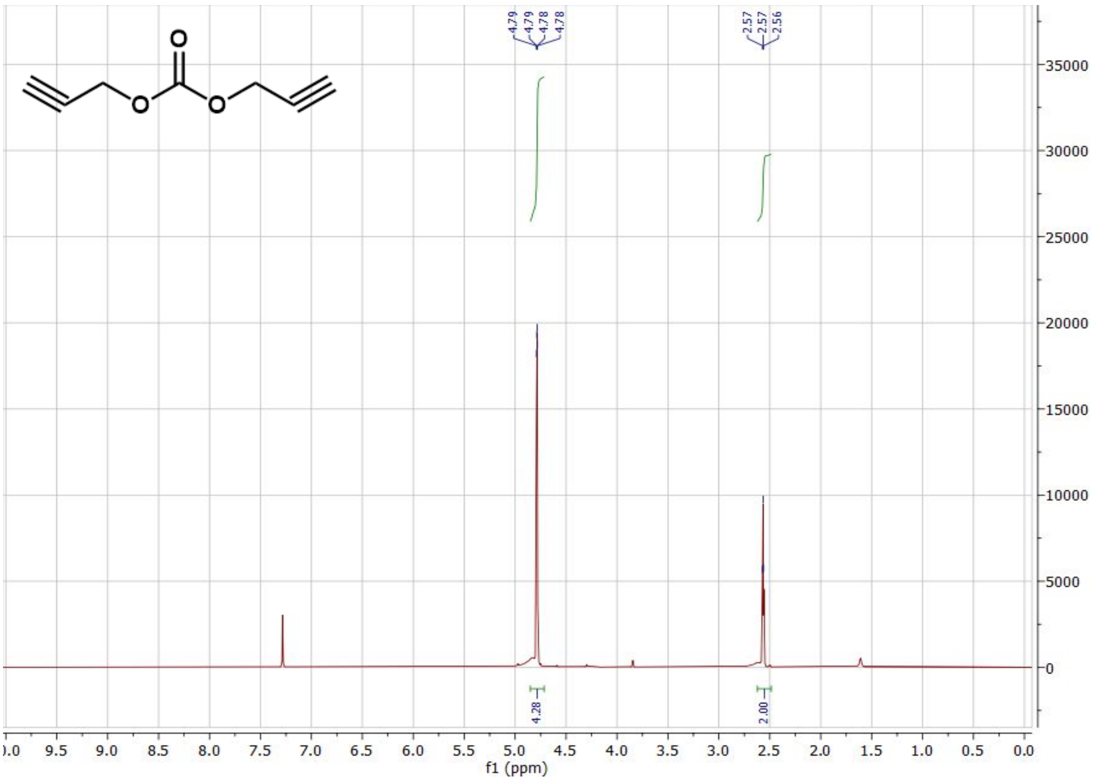

Figure S3: ^{13}C NMR of **dipropargyl carbonate** in $CDCl_3$

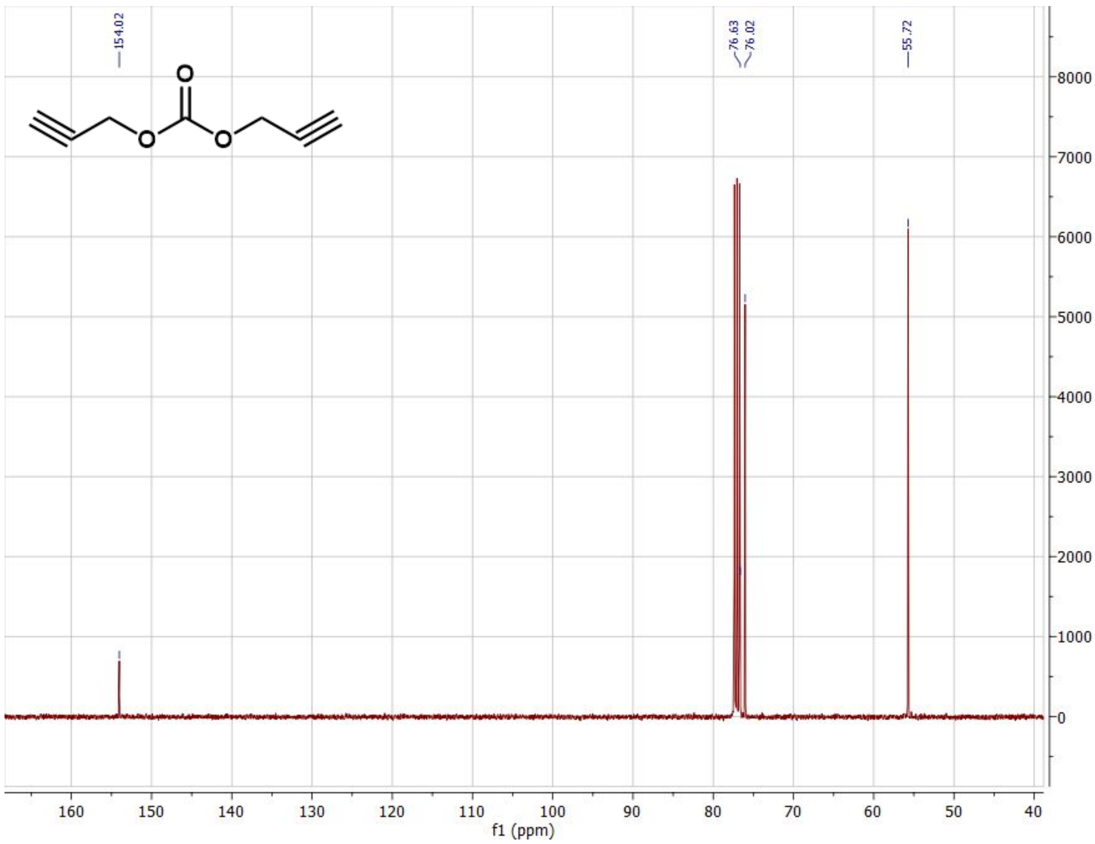

Figure S4: HRMS analysis of **dipropargyl carbonate** – Positive mode

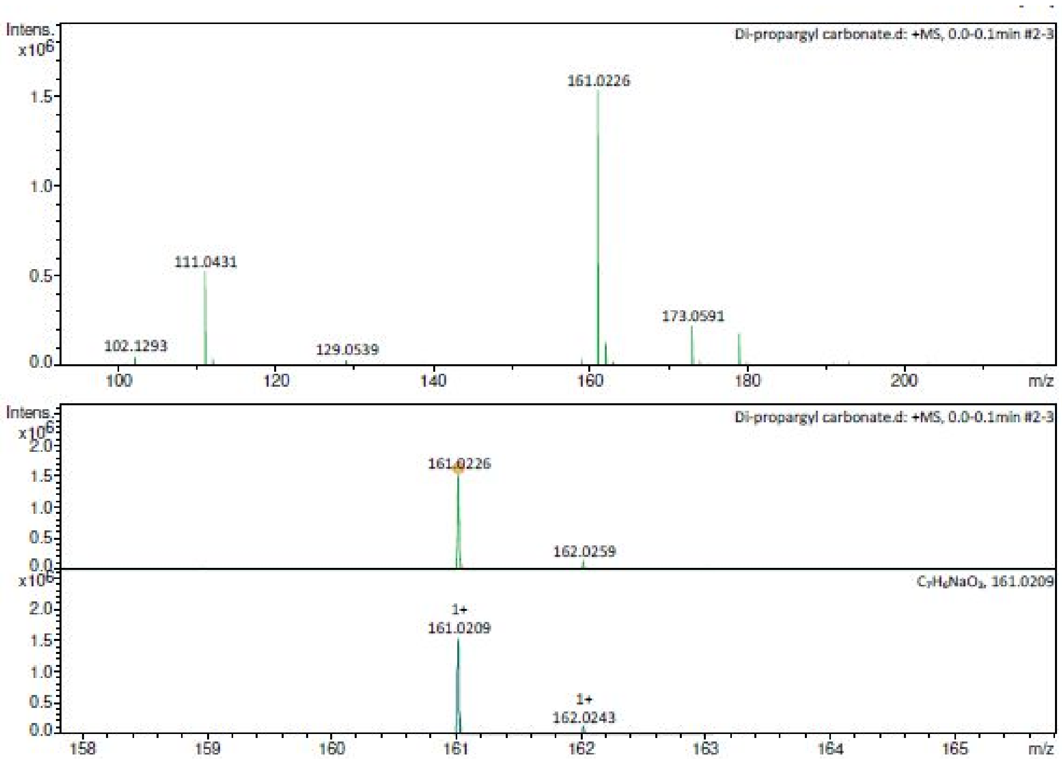

Meas. m/z	#	Ion Formula	m/z	err [ppm]	mSigma	# mSigma	Score	rdb	e⁻ Conf	N-Rule
161.0226	1	C7H6NaO3	161.0209	-10.3	3.6	1	100.00	5.0	even	ok

Analysis Info

Analysis Name	D:\Data\Jack\masse Arico 030625\Di-propargyl carbonate.d	Acquisition Date	6/3/2025 2:30:41 PM
Method	DirectInfusion - MS - positive.m	Operator	Demo User
Sample Name	Di-propargyl carbonate	Instrument	compact 8255754.20209
Comment			

Acquisition Parameter

Source Type	ESI	Ion Polarity	Positive	Set Nebulizer	0.4 Bar
Focus	Not active	Set Capillary	4500 V	Set Dry Heater	180 °C
Scan Begin	50 m/z	Set End Plate Offset	-500 V	Set Dry Gas	4.0 l/min
Scan End	1300 m/z	Set Collision Cell RF	650.0 Vpp	Set Divert Valve	Source

Figure S5: ^{1}H NMR of **isosorbide dipropargyl carbonate (IsDPC)** in $CDCl_3$

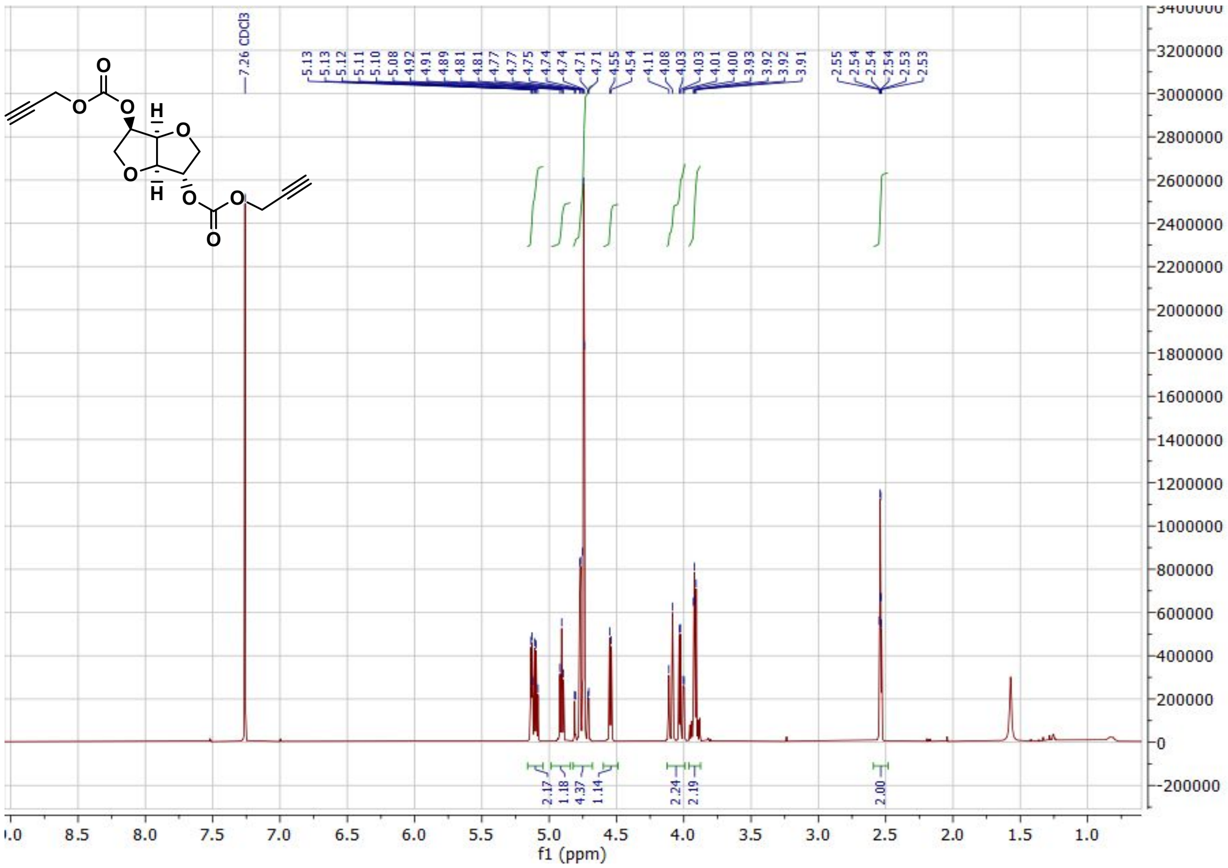

Figure S6: ^{13}C NMR of **isosorbide dipropargyl carbonate (IsDPC)** in $CDCl_3$

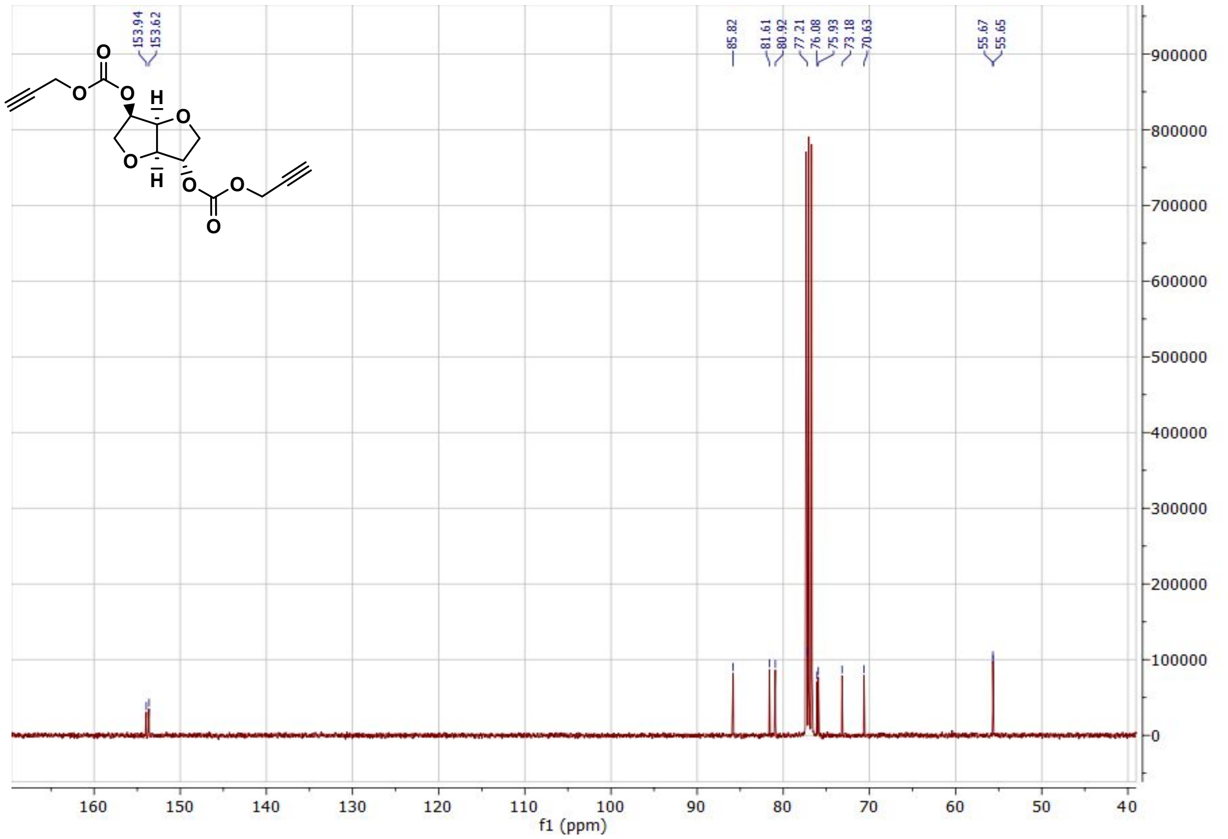

Figure S7: 2D NMR of **isosorbide dipropargyl carbonate (IsDPC)** in $CDCl_3$

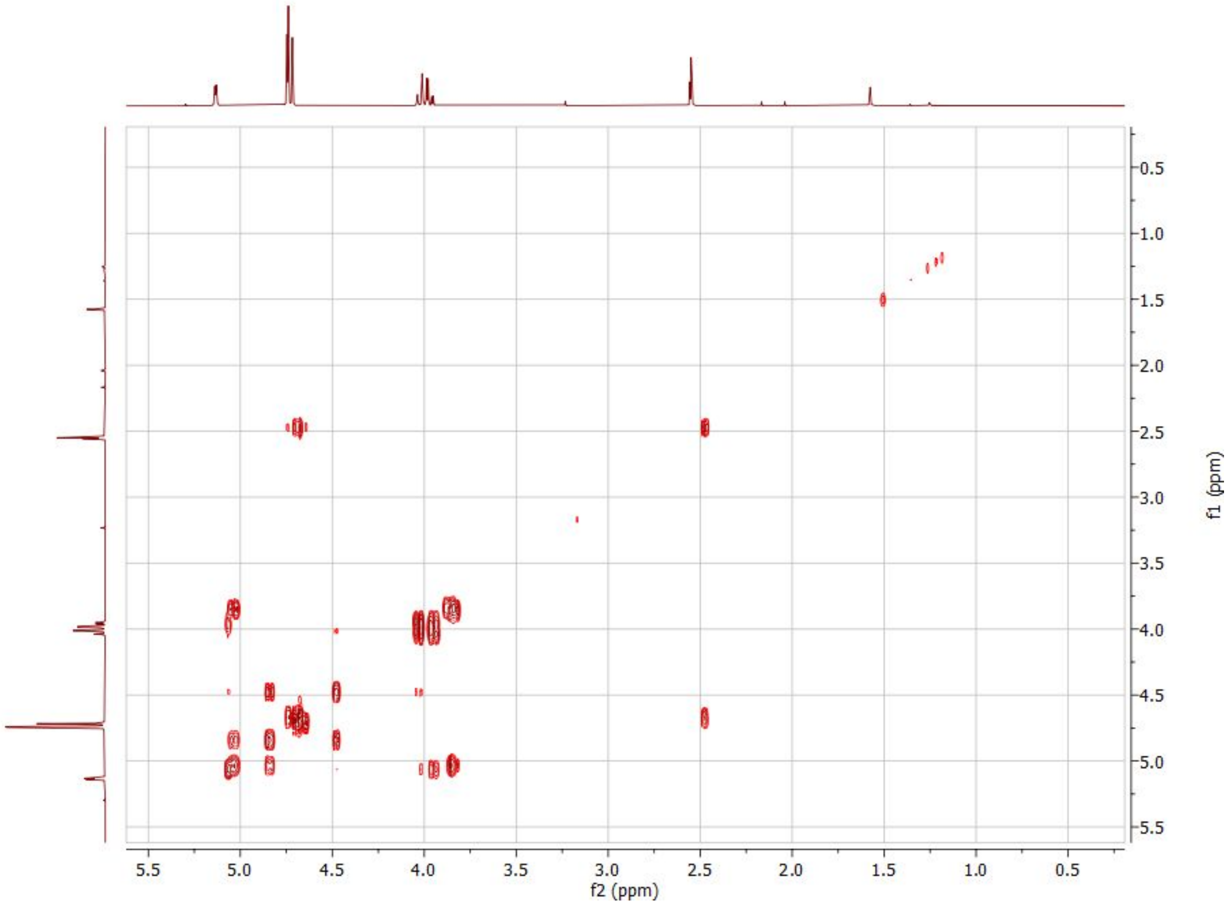

Figure S8: HRMS analysis of **isosorbide Dipropargyl Carbonate (IsDPC)–** Positive mode

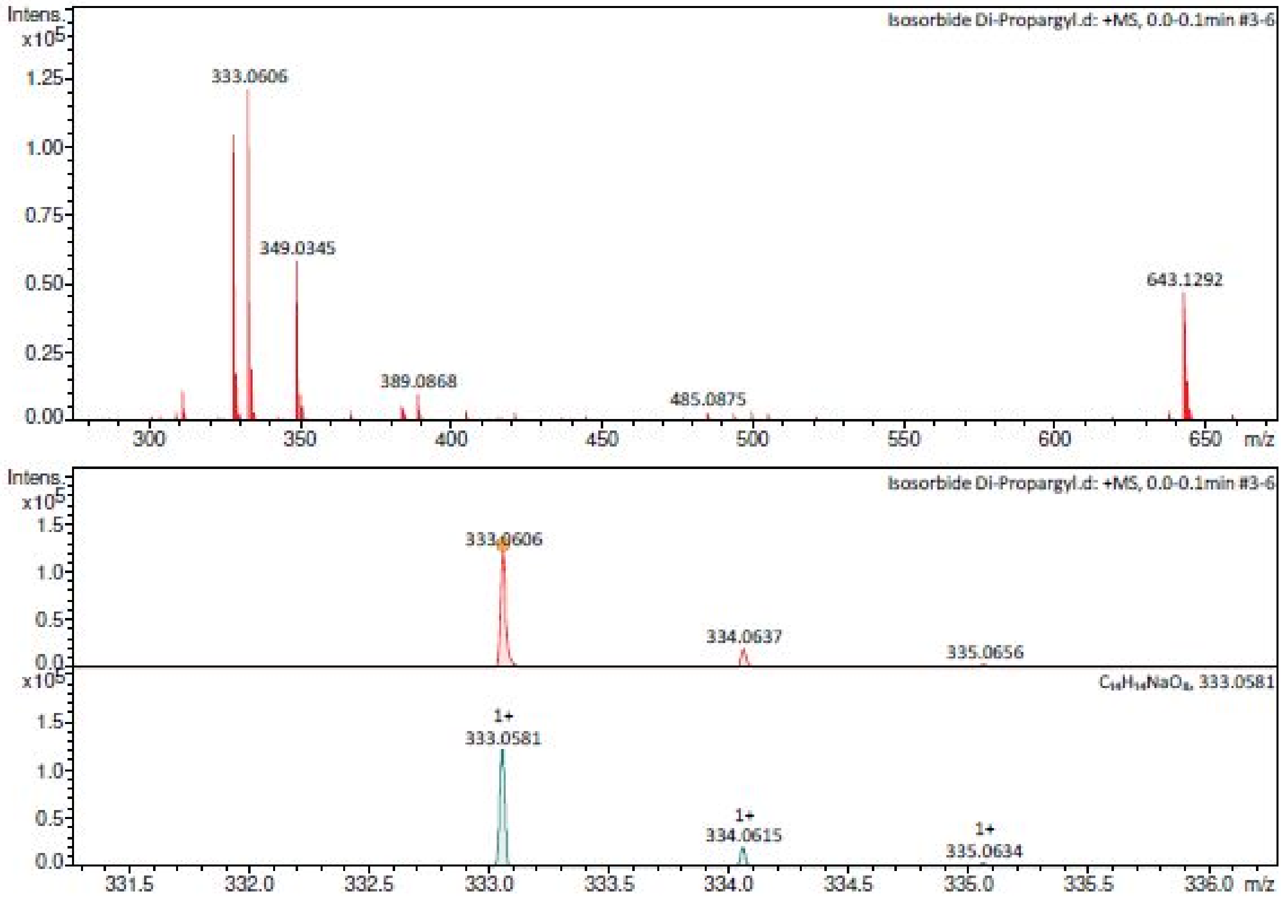

Meas. m/z	#	Ion Formula	m/z	err [ppm]	mSigma	# mSigma	Score	rdb	e⁻ Conf	N-Rule
333.0606	1	C14H14NaO8	333.0581	-7.6	1.0	1	100.00	8.0	even	ok

Analysis Info

Analysis Name	D:\Data\Jack\masse Arico 030625\Isosorbide Di-Propargyl.d	Acquisition Date	6/3/2025 2:49:24 PM
Method	DirectInfusion - MS - positive.m	Operator	Demo User
Sample Name	Isosorbide Di-Propargyl	Instrument	compact 8255754.20209
Comment			

Acquisition Parameter

Source Type	ESI	Ion Polarity	Positive	Set Nebulizer	0.4 Bar
Focus	Not active	Set Capillary	4500 V	Set Dry Heater	180 °C
Scan Begin	50 m/z	Set End Plate Offset	-500 V	Set Dry Gas	4.0 l/min
Scan End	1300 m/z	Set Collision Cell RF	650.0 Vpp	Set Divert Valve	Source

Figure S9: ^{1}H NMR of **isomannide dipropargyl carbonate (ImDPC)** in $CDCl_3$

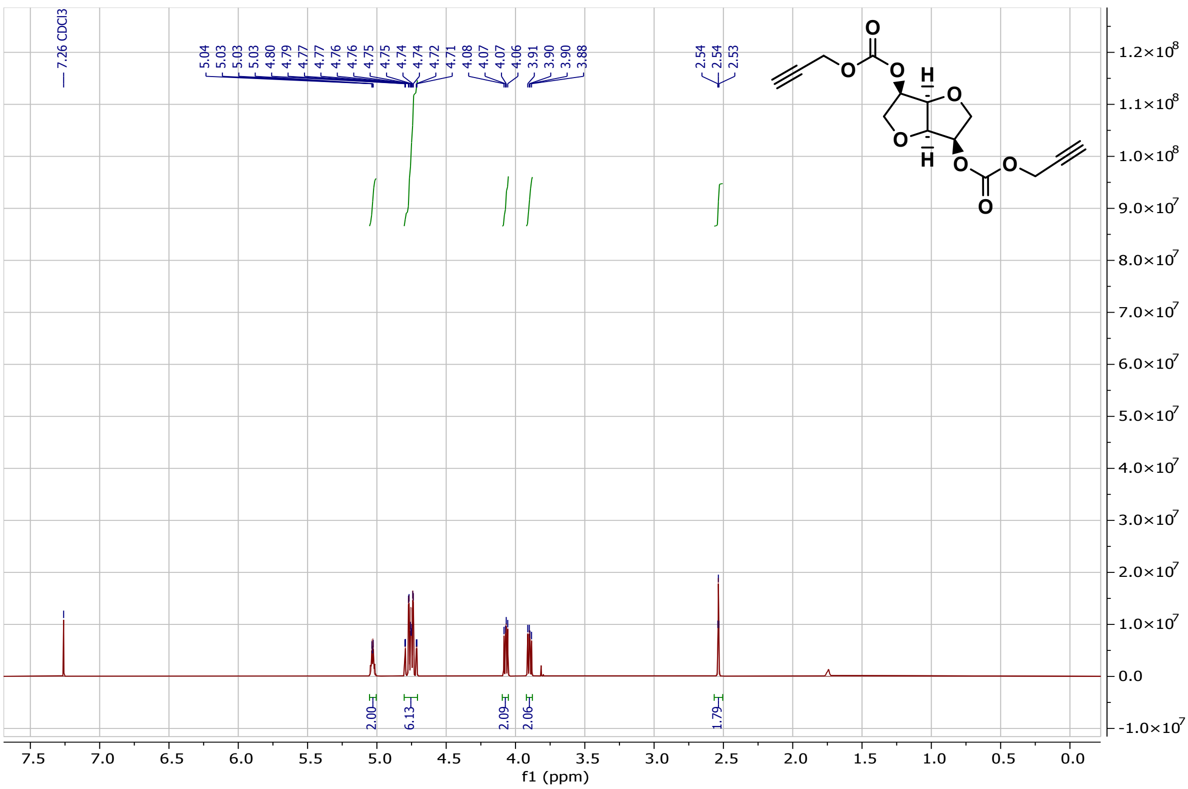

Figure S10: ^{13}C NMR of **isomannide dipropargyl carbonate (ImDPC)** in $CDCl_3$

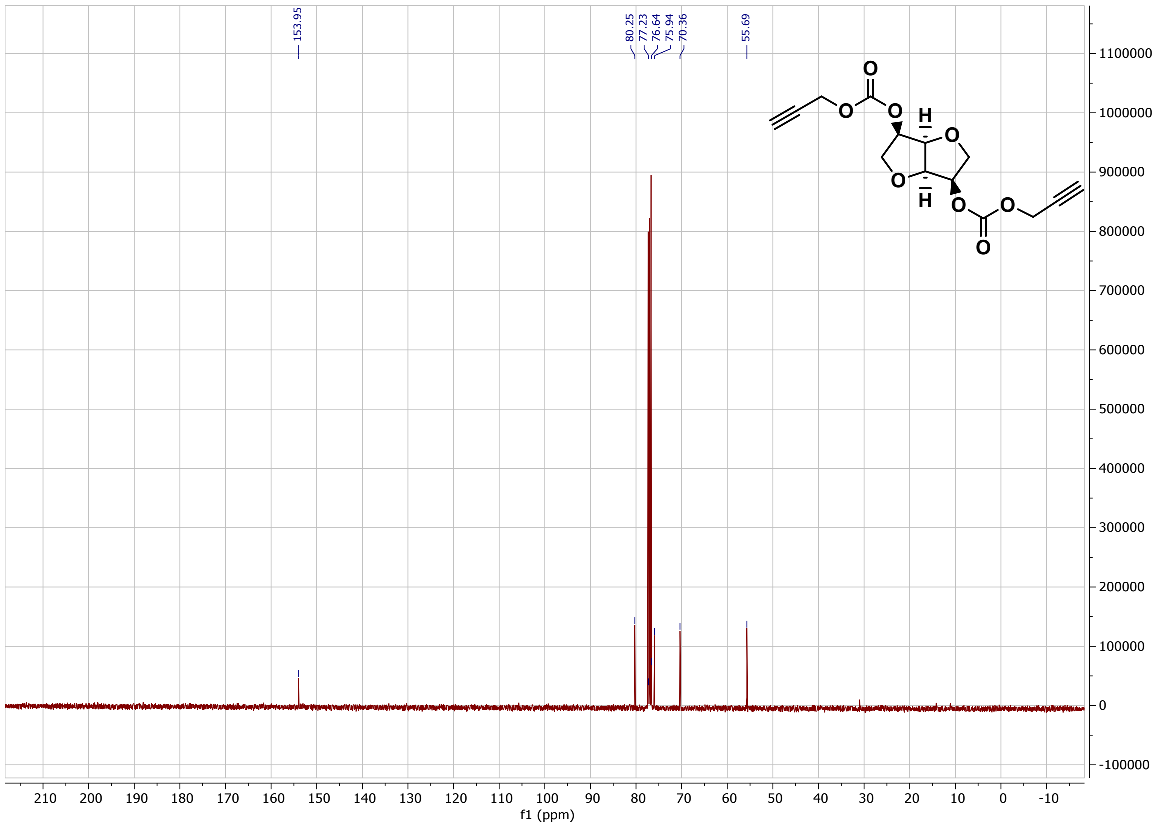

Figure S11: 2D NMR of **isomannide dipropargyl carbonate (ImDPC)** in $CDCl_3$

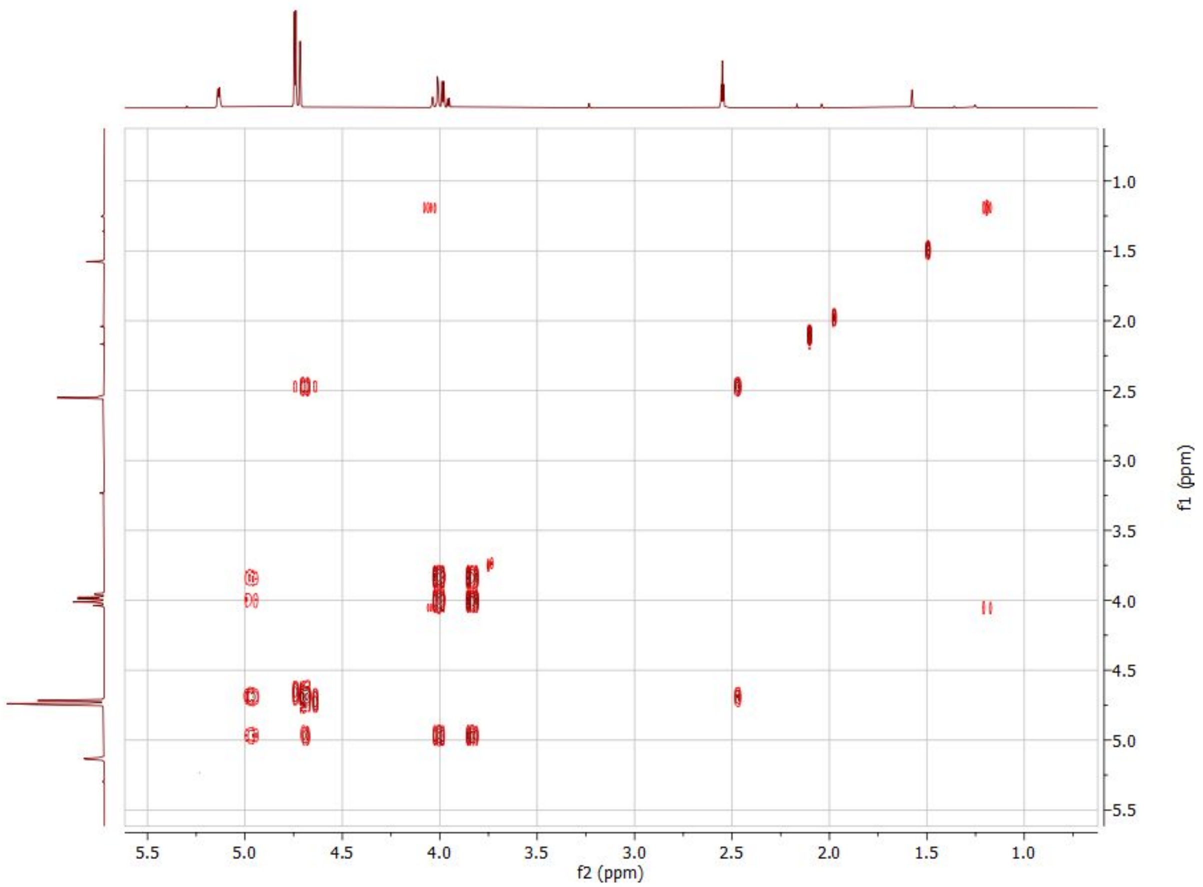
5.5
5.0
4.5
4.0
3.5
3.0
2.5
2.0
1.5
1.0
f2 (ppm)
f1 (ppm)

Figure S12: HRMS analysis of **isomannide dipropargyl carbonate (ImDPC)**– Positive mode

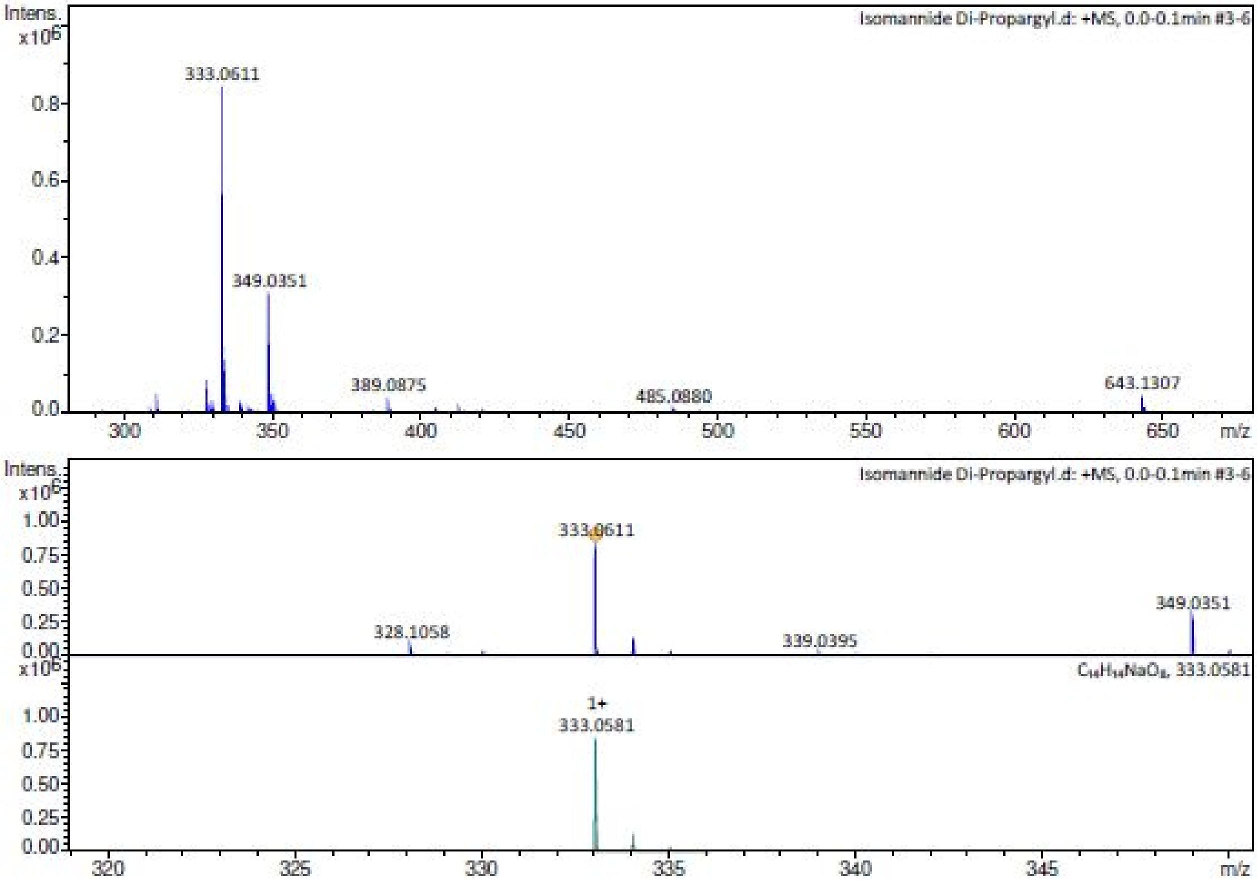

Meas. m/z	#	Ion Formula	m/z	err [ppm]	mSigma	# mSigma	Score	rdb	e⁻ Conf	N-Rule
333.0611	1	C14H14NaO8	333.0581	-9.0	3.1	1	100.00	8.0	even	ok

Analysis Info

Analysis Name	D:\Data\Jack\masse Arico 030625\Isomannide Di-Propargyl.d	Acquisition Date	6/3/2025 2:56:43 PM
Method	DirectInfusion - MS - positive.m	Operator	Demo User
Sample Name	Isomannide Di-Propargyl	Instrument	compact 8255754.20209
Comment			

Acquisition Parameter

Source Type	ESI	Ion Polarity	Positive	Set Nebulizer	0.4 Bar
Focus	Not active	Set Capillary	4500 V	Set Dry Heater	180 °C
Scan Begin	50 m/z	Set End Plate Offset	-500 V	Set Dry Gas	4.0 l/min
Scan End	1300 m/z	Set Collision Cell RF	650.0 Vpp	Set Divert Valve	Source

Figure S13: ^{1}H NMR of **isoidide dipropargyl carbonate (IiDPC)** in $CDCl_3$

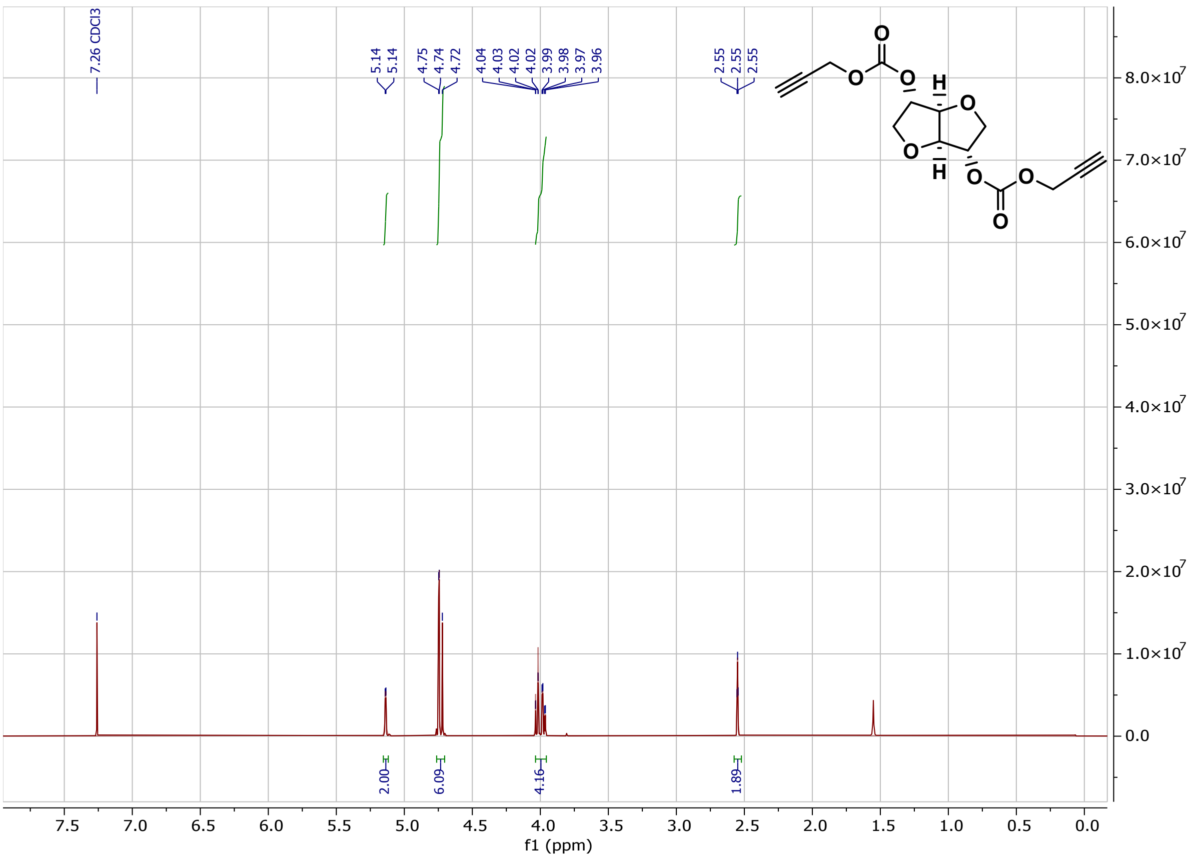

Figure S14: ^{13}C NMR of **isoidide dipropargyl carbonate (IiDPC)** in $CDCl_3$

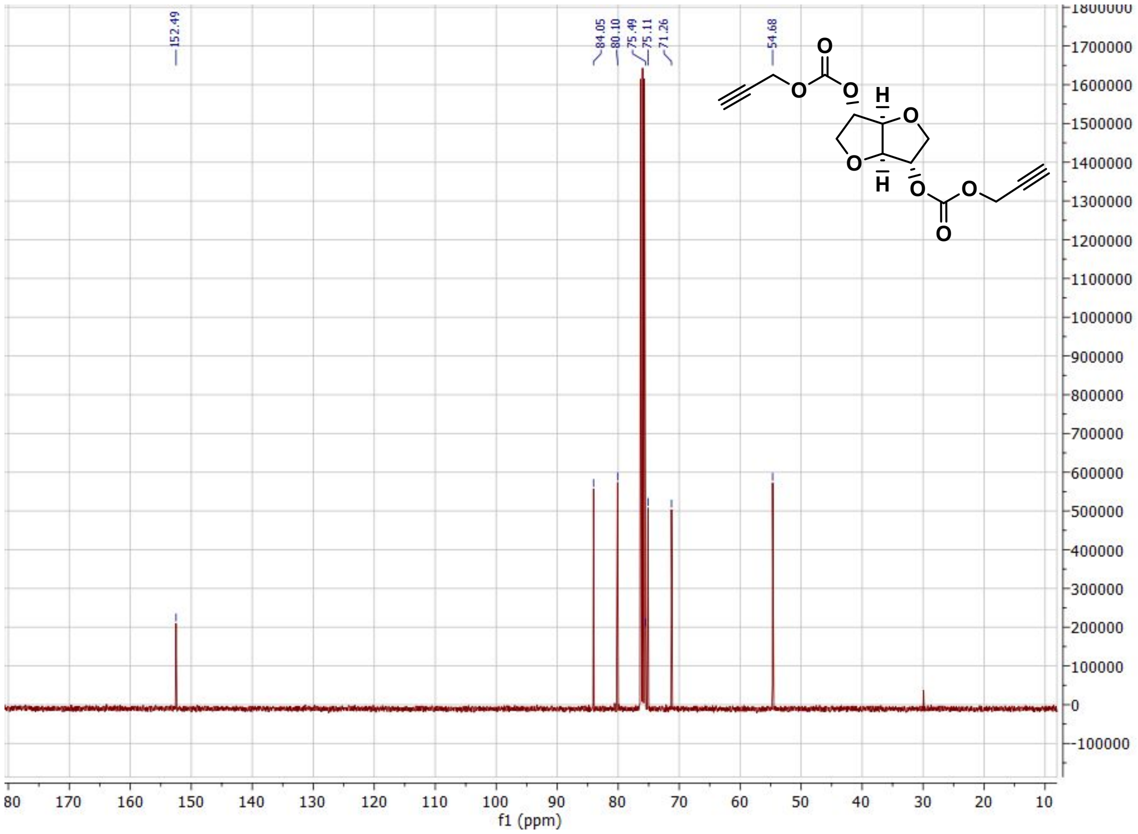

Figure S16: 2D NMR of **isoidide dipropargyl carbonate (IiDPC)** in $CDCl_3$

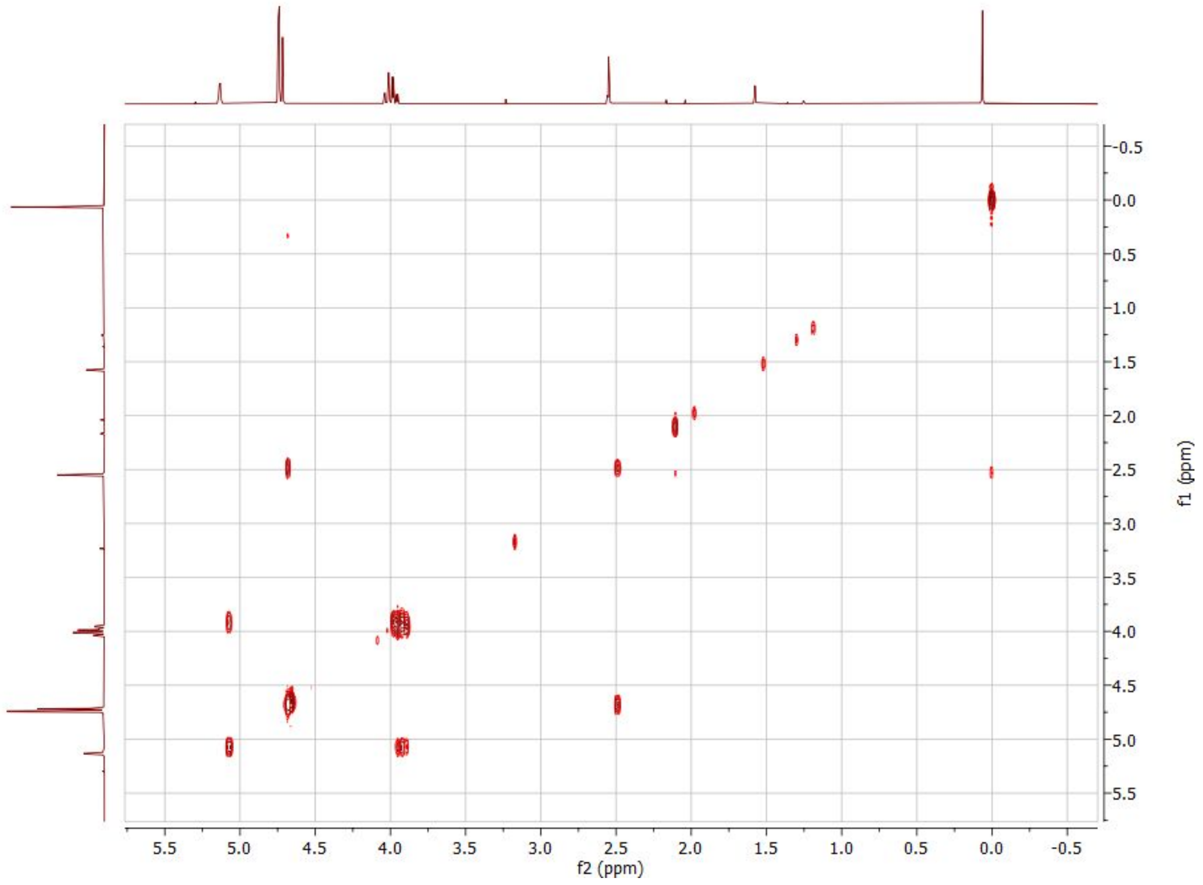

Figure S17: HRMS analysis of **isoidide dipropargyl carbonate (IiDPC)**– Positive mode

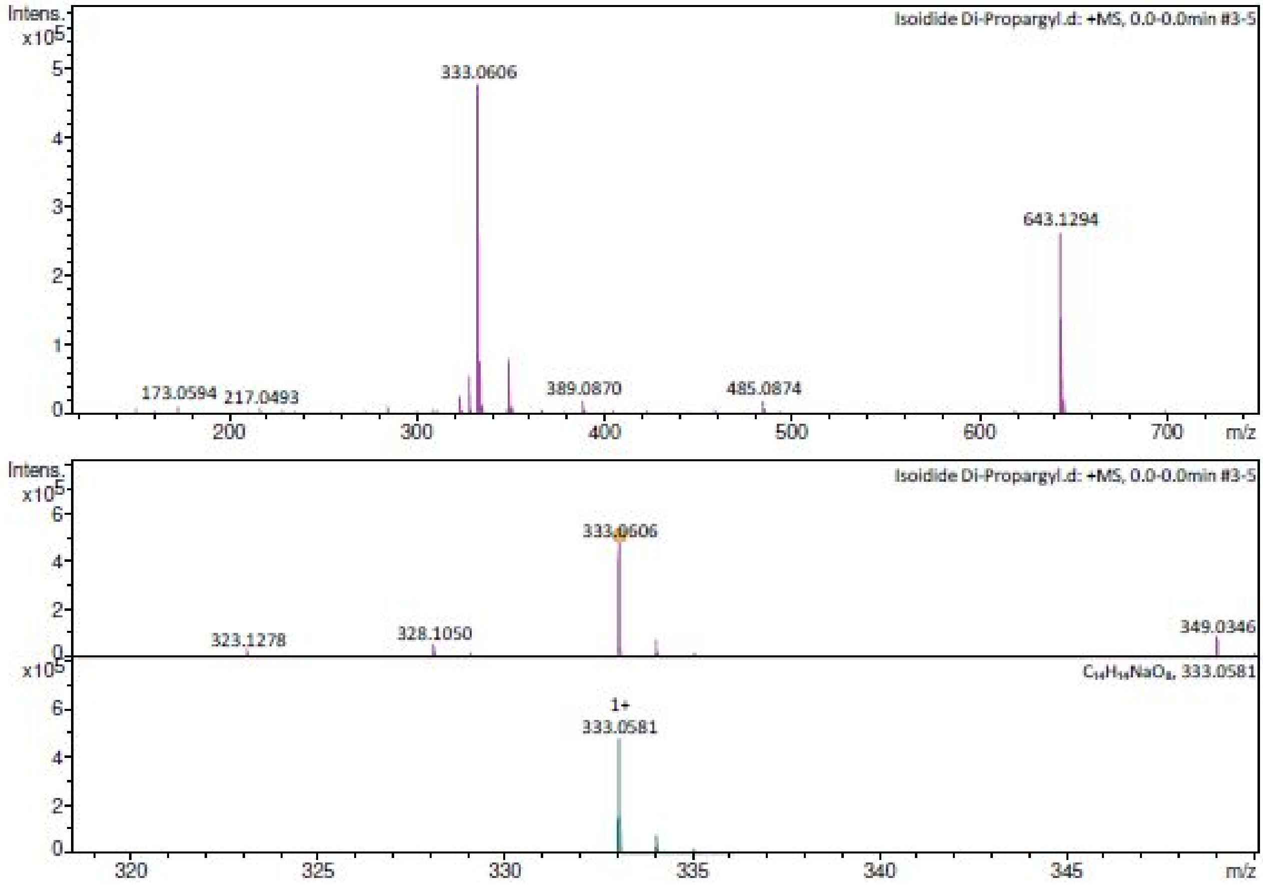

Meas. m/z	#	Ion Formula	m/z	err [ppm]	mSigma	# mSigma	Score	rdb	e⁻ Conf	N-Rule
333.0606	1	C14H14NaO8	333.0581	-7.6	2.7	1	100.00	8.0	even	ok

Analysis Info

Analysis Name	D:\Data\Jack\masse Arico 030625\Isoidide Di-Propargyl.d	Acquisition Date	6/3/2025 2:42:30 PM
Method	DirectInfusion - MS - positive.m	Operator	Demo User
Sample Name	Isoidide Di-Propargyl	Instrument	compact 8255754.20209
Comment			

Acquisition Parameter

Source Type	ESI	Ion Polarity	Positive	Set Nebulizer	0.4 Bar
Focus	Not active	Set Capillary	4500 V	Set Dry Heater	180 °C
Scan Begin	50 m/z	Set End Plate Offset	-500 V	Set Dry Gas	4.0 l/min
Scan End	1300 m/z	Set Collision Cell RF	650.0 Vpp	Set Divert Valve	Source

Figure S18: ¹H NMR of **isosorbide diallylcarbonate (IsDAllC)** in $CDCl_3$

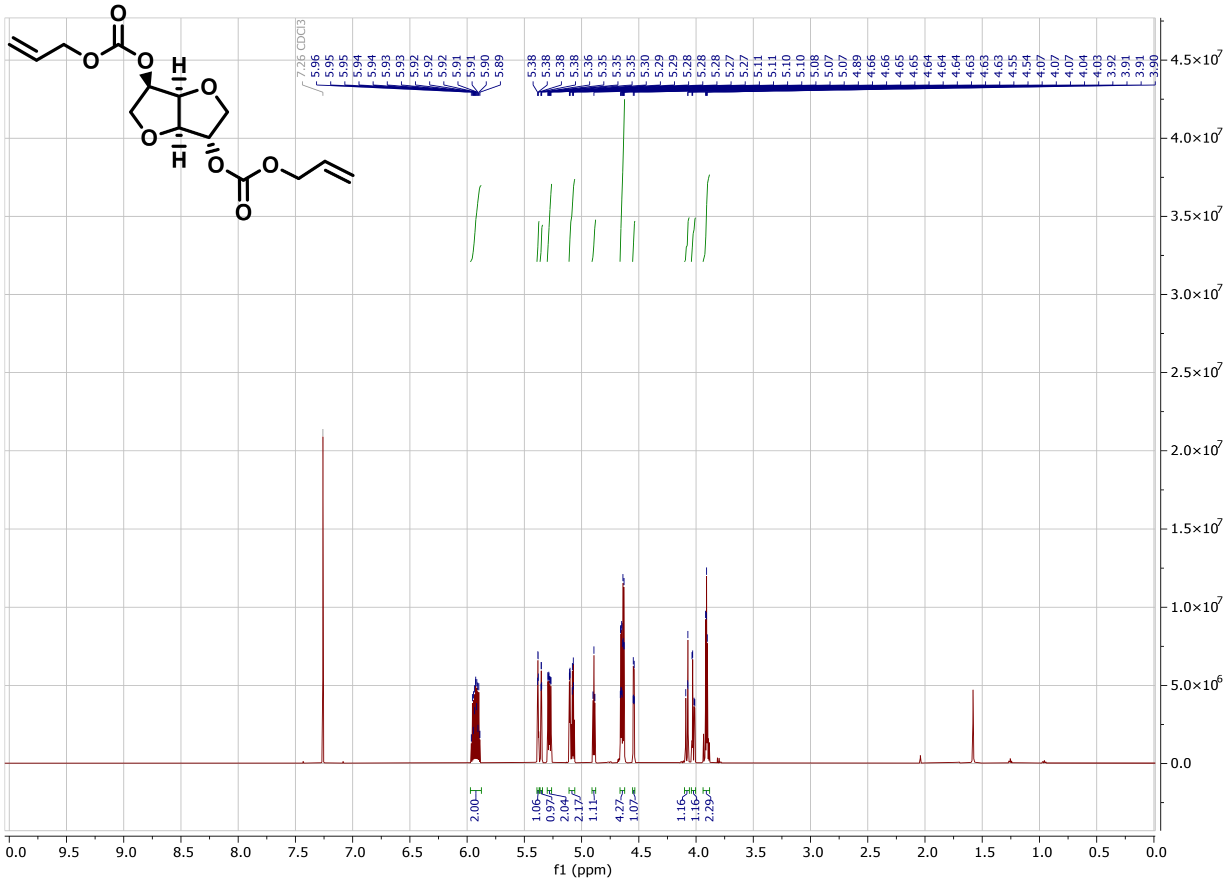

Figure S19: ¹³C NMR of **isosorbide diallylcarbonate (IsDAllC)** in $CDCl_3$

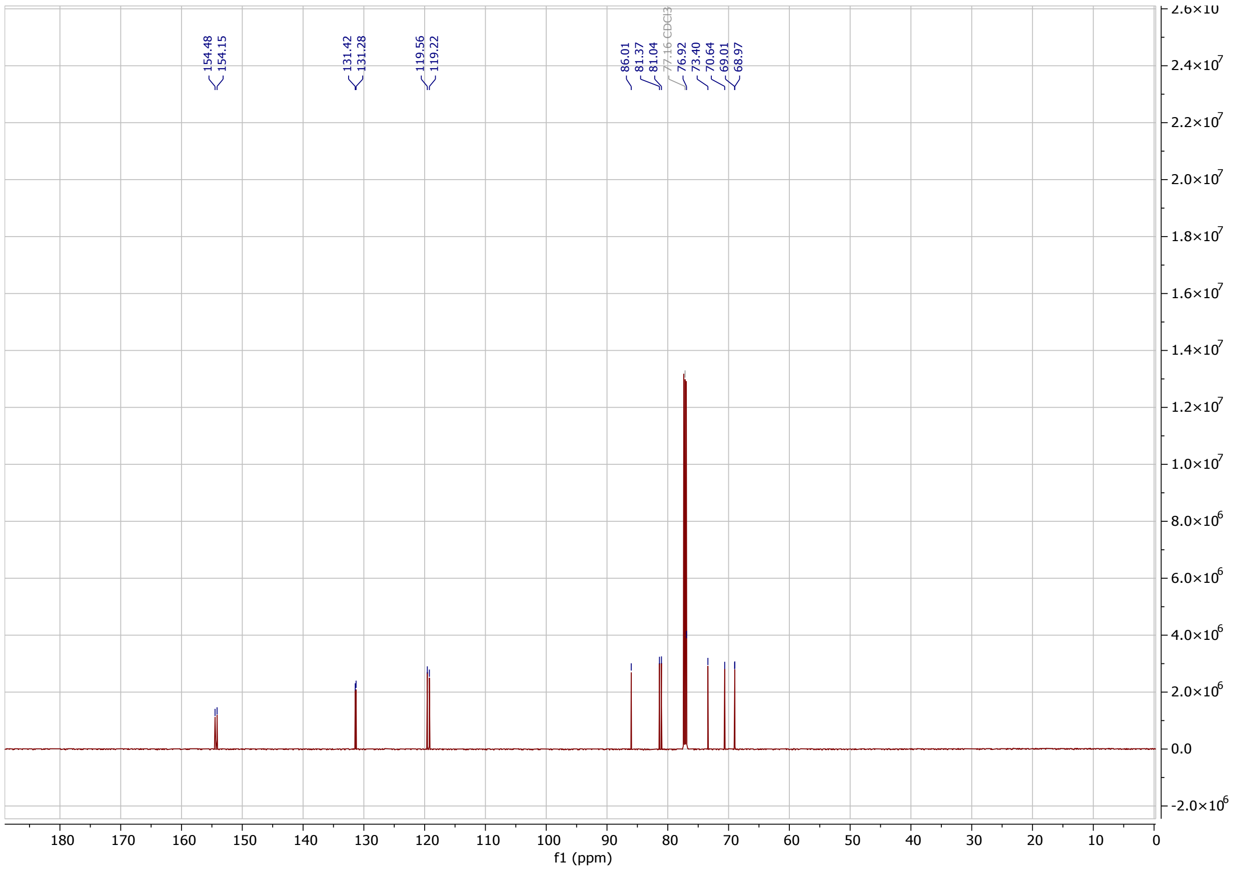

Figure S20: HRMS analysis of **isosorbide diallylcarbonate (IsDAllC)** - Positive mode

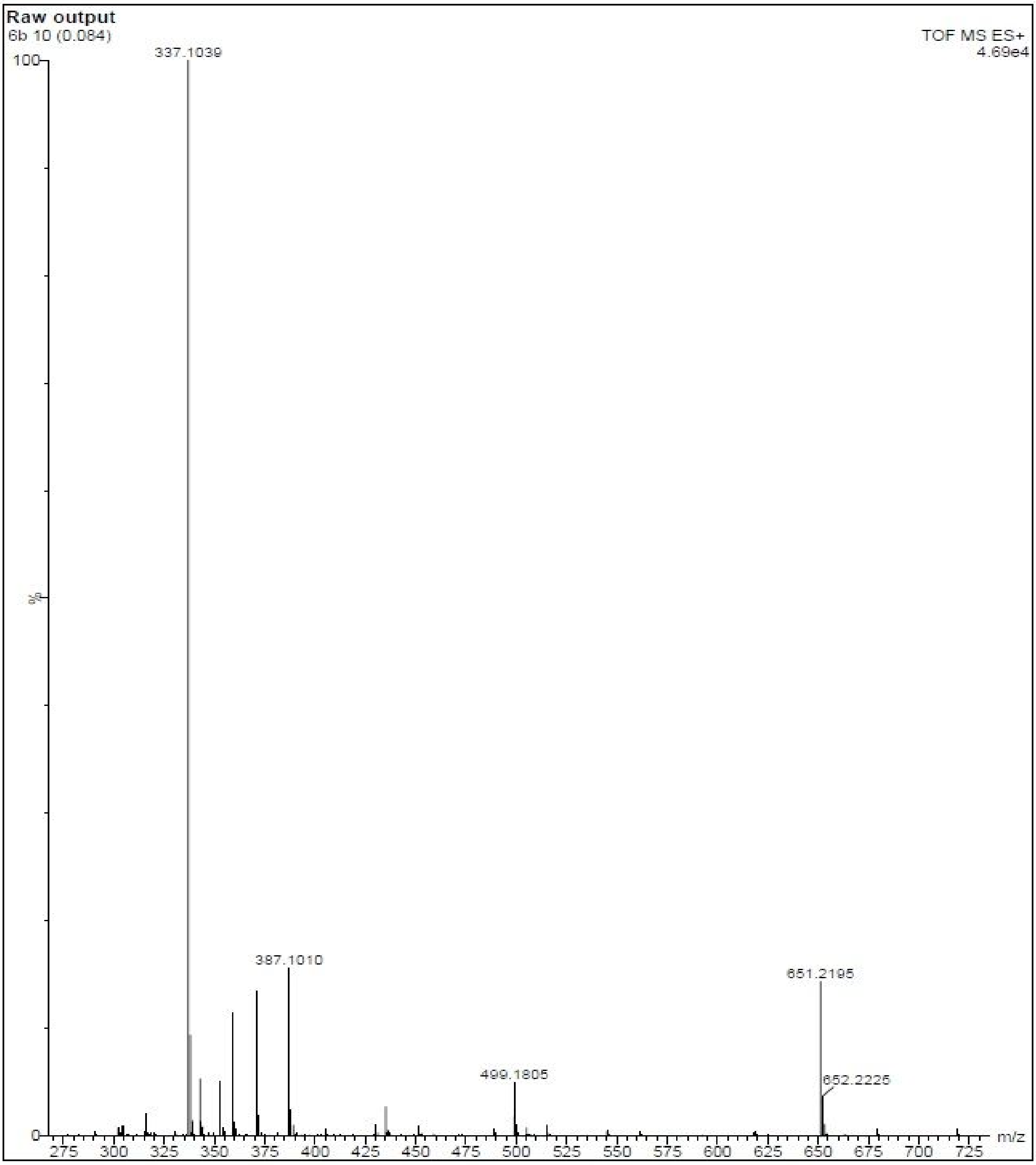

HRMS: m/z[M + Na]$^+$ calc. for $[C_{14}H_{14}O_8Na]^+$: 337.0894; found: 337.1039.
